# Interoperability architecture for data spaces

**DOI:** 10.1016/j.dib.2026.112678

**Published:** 2026-03-09

**Authors:** Juha-Pekka Soininen, Carlos Fernández Sánchez, Stefano Modafferi, Stuart Campbell, Noel Tomas, Eliot Salant, Christina Manara, Jarmo Kalaoja, Soumya Kanti Datta

**Affiliations:** a VTT Technical Research Centre of Finland, Kaitoväylä 1, 90570 Oulu, Finland; b Indra, Minsait, av. de Bruselas 35, 28108, Alcobendas, Spain; c University of Southampton, University Road, Southampton, SO17 1BJ. Southampton, UK; d Information Catalyst SL, C/ Reina 27, 7, Xativa, 46800, Spain; e IBM Research - Israel, University of Haifa Campus, Mount Carmel, Haifa 3498825, Israel; f Athens Technology Center (ATC), Rizariou 10, Chalandri 152 33, Greece; g Digiotouch, Narva mnt 5, 10117 Tallinn, Estonia

**Keywords:** Data sharing, Smart contracts, Data transaction, Data spaces

## Abstract

This paper presents an interoperability architecture for data spaces (DSIA), allowing members of the different data spaces using different technologies and services to exchange data. The DSIA builds on the existing trust of data space members and extends it through shared agreements and additional federation and participant services. The DSIA consists of four components: sub-systems for data sharing across data spaces, data pipeline, deployment, and a set of supporting applications for users. The DSIA has been validated with DS2 implementations and compared to other interoperability approaches. The main benefits are minimal independence to interacting data space technologies, little governance overheads, and easy deployment for existing data space participants and authorities’ systems.

## 1 Introduction

Today's society is based on Internet use, and data exchange is a key Internet functionality. Data can take various forms, such as media streaming, virtual reality, payments, and automation, making data valuable for providers and consumers [1]. The value of data stems not only from specific use cases but also from its sensitivity, confidentiality, or secrecy, which has led to various cybersecurity techniques to protect it. The need to share and protect data for value creation creates a significant conflict, as it involves concepts such as trust that cannot be fully addressed with technology alone.

The need to share confidential or sensitive data has made data rights and data rights holders an explicit concern. As data is easy to copy and its provenance is easy to hide, the full rights of data rights holders need protection using regulations such as the GDPR [2], the Data Act [3] and the Data Governance Act [4] in the EU. When such data is shared between legal entities, it is important to agree on precisely what rights are transferred and how data can be used, without violating the data sovereignty and the rights of the data rights holder. Practically, this means that in addition to the technical data transfer, the transfer of data rights must be considered, leading to legal agreements between the parties involved.

Data spaces have been proposed as a solution for sharing data between legal entities [5]. The key idea of Data Spaces is to create a trustworthy digital environment in which parties can publish and search for data offerings, agree on the conditions which the data can be exposed share the actual data, and, to some extent, also to monitor the use of data [6]. Data spaces aim to enable data productization to implement different business cases such as data marketplaces and value-creation networks [7]. Trustworthiness in a data space is established through a comprehensive framework that integrates identity management, role-based responsibilities, certified infrastructures, legal agreements, and robust governance mechanisms. These measures create data spaces for their members only, i.e., data silos, unless additional measures are implemented to ensure collaboration between individual data spaces.

This paper presents a data space interoperability architecture (DSIA) and its implementation in the EU Horizon DS2 project [8]. The data spaces can be implemented in various ways depending on the data-sharing needs. There are attempts to create common standards and to harmonise the data space concept, but the companies involved, and the markets will eventually decide what technologies and solutions are used. The DSIA approach minimises the federation between data spaces and provides dedicated services for interoperability. The DSIA is based on the idea that data exchange between members of different data spaces builds on trust created within each data space. This trust is extended through cross‑data‑space data‑sharing contracts. These contracts define data pipelines involving both parties.

Data space interoperability is significant because it enables complex value networks across domain or sector boundaries. It also supports the emergence of a digital single market for the data economy, aligned with the EU data strategy, EU Data Act, and Digital Single Market. The DSIA approach provides a value-adding solution that is easy to accept, deploy, and use for data spaces and their participants.

Data spaces and data space interoperability are new, emerging concepts [9]. The Catena-X and Smart Connected Supply Network interoperability analysis was reported in [10]. Common European Mobility Data Space has presented an interoperability proposal in [11] based on a common governance framework. From end-user perspective, the interoperability of data spaces allows to reuse the trustworthiness of the partner as a member of the data space in collaborations with members of the other data spaces. It introduces savings in terms of on-boarding evaluations and software infrastructures compared to joining to each data space separately. This paper provides an architecture and software solution for the interoperability of data spaces loosely coupled with the participating data spaces and a deployment solution with marketplaces and ecosystem models.

The remainder of the paper is organised as follows: Chapter 2 presents the related work with data sharing and data spaces and their interoperability challenges. Chapter 3 presents the data space interoperability architecture from functional system and ecosystem perspectives. Chapter 4 describes the DS2 software implementation and validation use cases. Chapter 5 discusses the results, and Chapter 6 gives conclusions.

## 2 Related Work

Data space interoperability has become important as the limitations of data spaces have become apparent [12]. Despite efforts to create a harmonised approach, it is not yet determined which approaches will be used in practical data-sharing use cases. A broader perspective on data sharing is needed, considering interoperability between participants involved in data transactions, leading to possibly overlapping concepts of data spaces and data space interoperability.

### 2.1 Data sharing and data spaces

Data sharing has been a foundational capability of the Internet, focusing on the technical capability to transfer bits and files [13]. As technology matured, it was possible to emphasise the needs of data transactions from confidentiality and business perspectives. The need for privacy and security led to cybersecurity [14] solutions to hide the data from external parties. The transition to the collaboration of companies and networked value creation created the need to manage data as assets with ownership and value and connected data management with business and legal processes [15].

Bilateral peer-to-peer (P2P) data sharing and data sharing via shared resources are the main approaches to sharing confidential data.

• The idea in P2P data sharing is that parties jointly agree on everything related to data sharing and using the technologies of their choice. The typical approach is that the data provider opens its API to the data consumer, and data transaction is agreed upon in separate contracts. The challenges relate to the scalability of the ecosystem [16].
• In the shared resources approach based on data platforms or data warehouses, the idea is that the data is stored in a single platform provided by a third party, and the platform provider offers solutions and processes supporting data sharing [17,18]. The benefit is a broader collaboration network through the companies that use or access the same platform and common services, such as data marketplaces, that make collaboration easier. The challenges are related to the third party, i.e., cost overheads and trustworthiness.

**Fig. 1:**
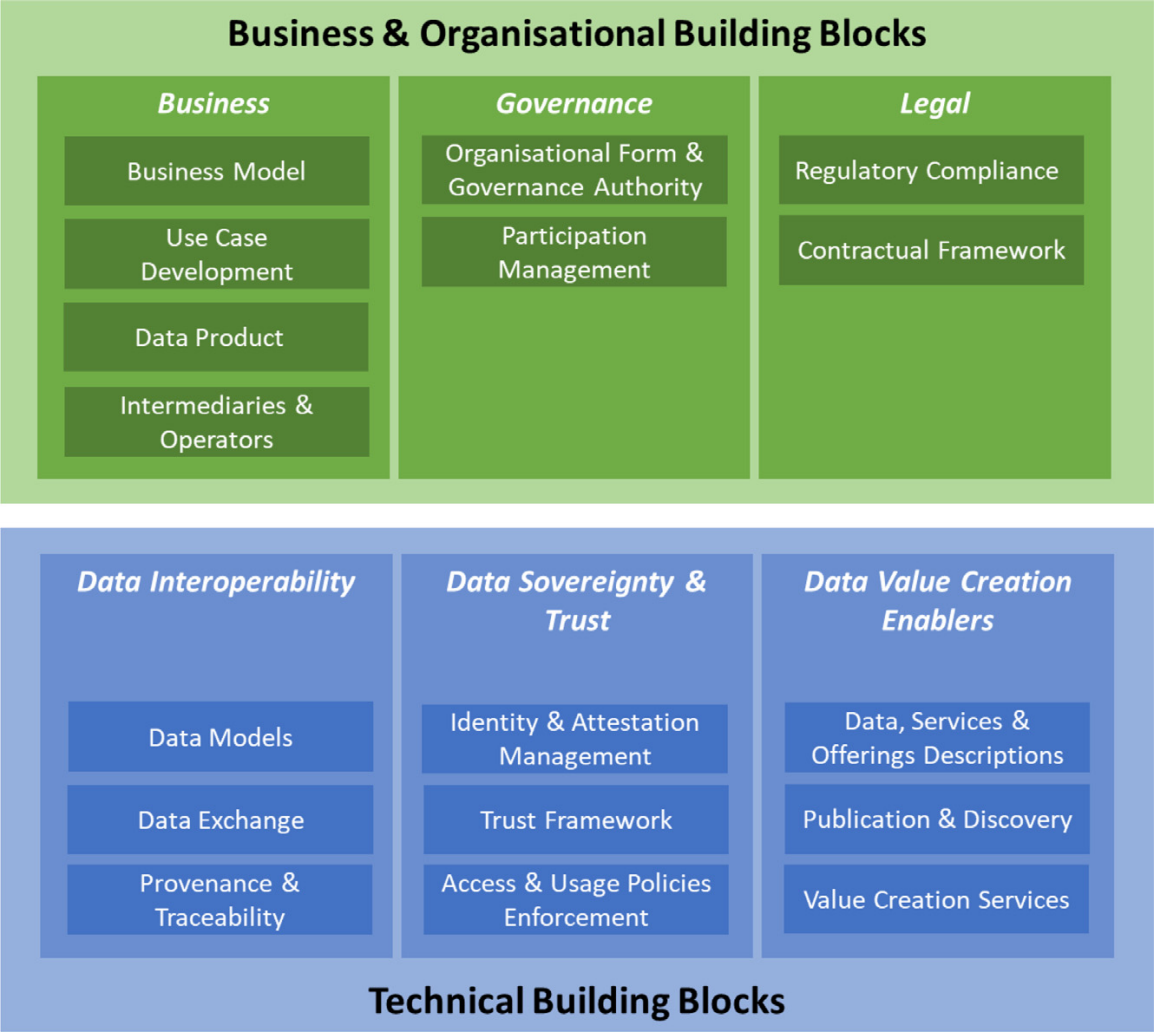
Data Space building blocks from DSSC [30].

**Fig. 2:**
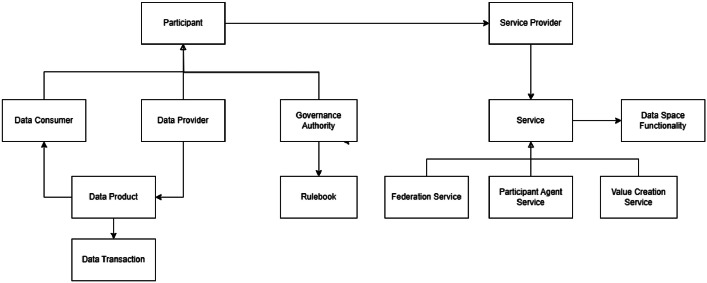
Data Space architecture from DSSC [30].

Data spaces extend the idea of bilateral data sharing with common trust creation services. The data space concept as an extension of data management with external data sources and a data offering catalogue was introduced by Franklin et al [19] and extended by Halevy et al [20]. The evolution of the data space concept concerning data management and distributed data access is given by Bacco et al [21]. The SITRA Rulebook [22] defines the principles of a fair data economy, linking business concepts to data sharing in the form of rules and data-sharing contracts. These were integrated with data spaces by Nagel and Lycklama in [23], where the division into technical issues and governance of data spaces was introduced. The development of data space specifications and reference architecture models has been driven by the International Data Space Association (IDSA) [24] and GAIA-X [25]. IDSA has developed a more centralised approach focusing on security and trust in networking and accessing data. GAIA-X has had a focus on creating a trust framework based on decentralisation. This work has been extended by the EU-funded Data Space Support Centre (DSSC) [26] in its Data Space Blueprint [27], aiming to define the design options for common European Data Spaces [28]. The DSSC has defined data space building blocks presented in Fig. 1 and an architecture model described in Fig. 2. The EU has also launched a SIMPL project that will create reference implementations for the main functionalities of data spaces [29].

The DSSC building block model (Fig. 1) divides data spaces into business and organisational building blocks (green) and technical building blocks (blue). Green blocks define how governance can be organised and what business-related issues must be considered. The legal part defines contractual framework and regulatory compliance issues. The green part focuses on definitions, methods, and processes. The technical blue part focuses on the technical specifications of core functionalities and data models needed. It is divided into three parts: data interoperability, data sovereignty and trust, and data value creation enablers. Even though the model is divided, the technical implementations from the blue part depend on the choices made in the green part. It is also important to note that the DSSC model is meant mainly for common European data spaces. Its objective is to describe the landscape from which each data space can pick the most suitable solutions.

The conceptual architecture of data spaces (Fig. 2) is a high-level and abstract presentation. On the left side of the diagram, there are **participants** who can have the roles of **data consumer, data provider**, and a **governance authority**, who is responsible for the **rulebook** that defines the operation principles of the data space. The data provider creates a **data product** acquired by the data consumer in a **data transaction** process.

The participant can be a **service provider** that provides **services** that implement **data space functionality**. There are three categories of services:

**Federation services** are related to data space operations:

• Membership management services such as onboarding, data space registry, off-boarding, financial sustainability services.
• Identity and access management services.
• Publication and discovery services for data product and service offerings.
• Logging services.
• Maintenance and operation services.

**Participant agent services** are for participant operations when using a data space:

• Contract negotiation and enforcement services.
• Data transfer services.

**Value creation services** are all the other possible services offered by data space. There are many types of services, and new innovations are expected to increase their number. The number of services available can also become huge.

### 2.2 Interoperability of data spaces

The need for data space interoperability has been obvious since the introduction of the common European Data Spaces concept, the definition of the first use cases for data spaces, and the definition of the first data space specifications. Use cases are diverse; they have different participants, needs for trustworthiness and data sovereignty, and data transfer characteristics [31].

The potential need for synergies and interoperability of data spaces was analysed in DSSC Synergy paper [32]. The outcome was that built-in capabilities for interoperability would be ideal, but the diversity of use case needs and rapid technical development will create a big challenge for it. The common European data spaces are large sectoral initiatives and data spaces that will most likely consist of multiple smaller data spaces requiring interoperability solutions. As a result of the efforts of the data space community, the cross-data space interoperability is defined by DSSC [27] as:

“The ability of participants to seamlessly access and/or exchange data across two or more data spaces. Cross-data space interoperability addresses the governance, business, and technical frameworks to interconnect multiple data space instances seamlessly."

The DSSC data space model gives freedom to develop different types of data spaces. Examples include co-creative approaches based on a single specification but multiple use cases and implementations [33,34], data spaces as service approaches [35,36], and platform-based approaches [37], where a data management platform and a data marketplace are extended to a data space.

**Fig. 3:**
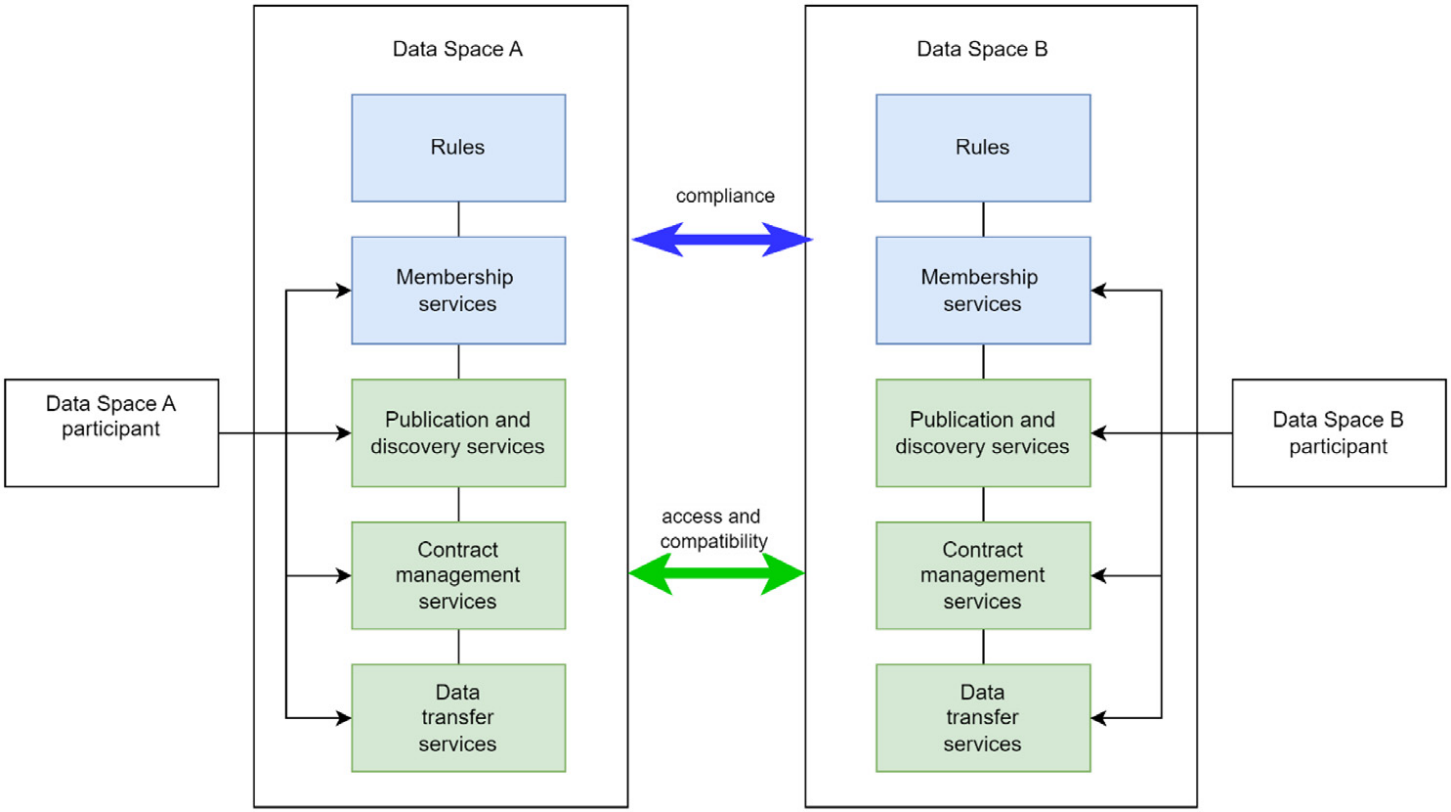
Data spaces' interoperability challenges.

Fig. 3 outlines the main challenges when two data spaces interoperate. From the governance perspective, compliance with the rules of data spaces concerning interoperability level and objectives needs to be agreed upon and implemented. The services and functionalities of data spaces need to be interoperable and there needs to be an agreement between data spaces to trust each other. As the number of data spaces is expected to increase, the scalability of data space interoperability solutions becomes quickly important.

The first key issue is extending the trust across data spaces. Within a data space, trust is created through known and verifiable identities, commitments to common data space rules, use of commonly accepted technical solutions, and commitment to common legal frameworks. All these aspects are defined in a data space rulebook, which is enforced by legally binding agreements between the governance authority of the data space and all its participants and service intermediaries, as well as by technical means such as verifiable certificates. In the inter-data space use case, the trust creation needs to be based on data spaces' mutual agreements and interoperability of technical capabilities related to trustworthiness.

The second main issue is data sovereignty, which states that the data rights holder must have full control over the use of its data. Full control means that during the data transaction process, the data rights holder must be able to control both the visibility of the data offer and the use of data.

**Fig. 4:**
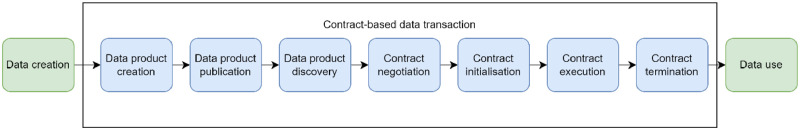
Contract-based data transaction process [38].

In practice, data space interoperability means that members of different data spaces can exchange data through contract-based transactions. The contract-based data transaction process described in Fig. 4 is essentially the same as in intra-data space use cases. The challenges arise because data spaces use different governance models and technical solutions.

The data transaction process consists of phases done by the data provider, consumer, or both. In this paper, the focus is on the requirements of inter-data space transactions. The main technical requirement is the ability of data space participant systems to communicate with each other. There must be common communication protocols for publishing data offerings and discovery, contract negotiation, contract executions, logging, and data transfer. Secondly, there must be capabilities to map different data formats and models so that both sides of the transaction can understand them. Thirdly, the data spaces may have different processes for the different stages of the transaction. These processes must be aligned. The transaction flow is as follows:

1. The process starts with the creation of data products and data product offers. The inter-data space operation sets extended requirements for defining the visibility of data product offerings.
2. Data product offer publication the offering is exposed to targeted data consumers.
3. During the data product discovery, the service exposing the data product offerings must be able to present the results so that they are understandable to potential data consumers and filter out those results to which the consumer has no or limited rights.
4. When negotiating contracts, either a service translates the contract terms between data spaces, or the data spaces use a shared contract model.
5. Contract initialisation means setting up the necessary services and the data pipeline between participants. Services include, for example, a contract execution engine, logging, and data access services. The data transfer pipeline may involve functionalities such as format transformations, anonymisation, and data quality validation.
6. Contract execution means parallel, coordinated execution of contract conditions by both participant systems. Contracts include policies that can be enforced within the data space's participant services, but there can also be services that need external enforcement solutions. Both participants must accept these solutions.
7. Contract termination is done similarly to intra-data space contracts.

Monitoring policies during data use is the most challenging part of data sharing. If monitoring by data space infrastructure is required, it can only be done when the data remains in a trustworthy environment. This means that the application using the data must be brought into the data space as a trustworthy application. The IDSA reference architecture [24] describes an app store concept and a runtime environment for the applications. When data leaves the trustworthy environment, the control of the data use can be made only using indirect methods, and the detection of possible misuse needs to be solved either at governance levels of interoperability solutions providers, data space providers, or through legal authorities.

### 2.3 Usability of data

Data and data sharing are limited if the user cannot understand the data, or the data does not meet the user's quality criteria. These issues depend on the creation of data and fall under the responsibility of the data provider and there are many possibilities for processing the data as part of the data transaction to improve its usability.

The idea in data spaces is that the data provider prepares its data as a data product accessed through a data service by a data space connector when the data sharing contract is executed. When the objective of a data provider is to maximise the use of its data product, this can lead to a situation where data consumers may require a huge variety of data formats, data models, used semantic models, and quality parameters. Providing all this variety beforehand, during data product preparation, is costly, technically difficult, and it may be wasteful if such consumers do not exist.

Data management systems have different services such as data transformations, data curation, data validation, quality evaluation, anonymisation, filtering, etc. These can be used for the creation of modified data that fits better for intended use or gives more certainty for the data user that the outcome of its data use meets the requirements [39]. For more complex data processing sequences, there is the concept of a data pipeline, connecting multiple services, which needs to be supported with tools for design and execution [40]. The idea has also been proposed for data spaces in the IDSA reference architecture in the form of applications uploaded from the IDSA app store and executed inside the IDSA data space connector equipped with a runtime environment [24]. The IDSA approach can also be used for apps that analyse data and transfer only analysis results to the data consumer. This allows the use of original data without exposing it to users outside the trustworthy environment of the provider. The benefits of connecting data processing capabilities to a data space are that it reduces the work of the data provider and increases the number of potential consumers. It makes the data product more usable and flexible, and services can create additional value through, for example, validated quality. The drawbacks are the cost of services, their coordination, and the additional effort needed to design the data pipeline during the contract negotiation phase.

### 2.4 Standardisation

Compatible interfaces and frameworks directly support frictionless data sharing across data spaces and adopting common standards is a straightforward way achieving this. The most relevant existing standards relate to data models used also outside data spaces. For example, the W3C Data Catalog Vocabulary Application Profile (DCAT-AP) [41] is a standard approach for presenting the meta-data of data offerings and participant descriptions. The Open Digital Rights Language (ODRL) [42] is a policy description language proposed for data sharing contracts. Both these standards provide a basis for data models, but the actual models should be defined more accurately by data spaces and/or participants, before interoperable systems can be constructed. W3C Verifiable Credentials and Verifiable Presentations (VC/VP) data and ecosystem models [43] are proposals for identity and access management.

Standardisation of data space technologies is in progress. The standardisation of key terms of data spaces and transaction process has been started in CEN CENELEC Workshop Agreement in Trusted Data Transactions [44]. The Data Space Protocol is being standardised in ISO JTC1 [45]. The CEN & CENELEC Focus Group (JTC25 WG2) on “Data, Data spaces, Cloud and Edge” aims at enabling seamless cross-sector data exchange, interoperability and trust covering data interfaces, metadata models, and security protocols, and strengthening European digital ecosystems by harmonised standards.

### 2.5 Interoperability concepts

According to the authors' knowledge, there are no published implementations of interoperability for data spaces, but various interoperability concepts have been presented, for example, in DSSC. The three main categories of concepts are federated data spaces, collaborative data spaces, and the use of intermediaries that provide interoperability services for data spaces.

The basic ideas behind these concepts differ, even though they all aim to implement the same functionality. The core differentiator is how federation services for interoperability and the definition of policies applied to inter-data space data transactions are implemented and coordinated.

• In **federated data spaces**, the idea is that a governance structure above all the participating data spaces provides means to access the services implemented by data spaces. The governance authority also defines the policies used.
• In **collaborative data spaces**, the data spaces implement the additional services needed for interoperability and define policy agreements. In [46] this is called an inter-linking layer.
• In **the intermediary concept**, the service provider provides services and mechanisms for agreeing on inter-data space policies.

In all concepts, participants must share a common technology for inter-data-space operations. It means either a separate connector and common data space protocol, or the capability to translate protocol messages between the solutions used in data spaces. Every concept also needs to implement a similar solution for data product and service discovery that is accessible to all participants involved in inter-data space operations. Otherwise, the participants cannot find the data offerings or federation services across data space boundaries.

The main benefits and challenges of each concept are as follows:

• Federated data space benefits from using the services of each data space. If the solutions within the federation are similar, e.g., in GAIA-X-based data spaces, this is a clear benefit, as access can be controlled using separate verifiable credentials only [47]. If the participating data spaces use several technologies, the situation becomes more complex. Federation requires additional standard federation services, data, and policy models, or additional capabilities for participants to access services in other data spaces. The outcome will be another data space on top of participating data spaces, with all the additional services and the duplication of effort needed by participants.
• In collaborative data spaces, the inter-linking layer implements the interoperability-by-design principle. The benefit for participants is that the required capabilities are built into the data spaces themselves. The interlinking layer increases the complexity of the participant agent services by introducing an additional data space connector. The data space must manage the collaborations and policies with all the other data spaces in the network, which may lead to complex processes. The interlinking layer also needs additional standard services for networking data offerings and participants, as well as someone to manage them.

The intermediary concept's main benefit is that it does not require any changes to the data space's own solutions. Everything is used as a service. It has two main options. First: (1) **Interoperability is provided by an intermediary** so that data space participants and data space services are **connected using data space's own solutions**. For the participant and the data space, this is the simplest solution, as no changes are required for either. The main drawback is that point-to-point data transactions between participants are unavailable. The data itself must go via an intermediary due to technical incompatibility between the provider and the consumer. Another drawback is that the intermediary must transform all messages and data formats between participants, resulting in a complex, expensive implementation. The second option is that (2) **the intermediary provides standard solutions** to all participants that use its services. In this case, the participant can operate in inter-data space activities as a member of its home data space but use similar participant agent services in trustworthy data transactions. The intermediary provides publishing, discovery and access control services. The main benefit is that no changes are needed to the data space itself, and data transactions are like point-to-point operations in a normal data space. The drawbacks are the need to deploy additional participant services and to integrate them with participants' data management solutions.

## 3 Research Method

The constructive research method [48] has been applied in this work with some adaptations due to the nature of the interoperability solution, as the interoperability solution is not a complete, stand-alone system and cannot be constructed and analysed. Interoperability solutions must interact with data space federation services and participants, which requires adaptability and flexibility. In this research, the interoperability problem has been analysed, a software system architecture to solve the problem has been designed, prototype implementations of core components have been developed, the feasibility of the approach using use case scenarios has been evaluated, the approach has been compared to other proposed interoperability concepts, and the main features of our approach has been assessed.

## 4 Data space interoperability architecture

The Data Space Interoperability Architecture (DSIA) presented in this paper is based on having a data space intermediary that hosts the federation of services the participants need to share data across data spaces and that provides needed participant services for them. The aim is to create an ecosystem where data spaces (represented by their governance authorities) and their participants can join and agree with other data spaces on data sharing. The ecosystem and approach also include support for users to manage the complexities of data transactions through extendable supporting functionalities. In this chapter, a system-level architecture is presented.

### 4.1 Principles of data space interoperability architecture

The main principles of the inter-data space interoperability approach presented in this paper are:

• Trust creation between parties performing the data sharing must be based on their trustworthiness as participants of their data spaces. Trust is composed of complementary aspects of hard and soft trust [49] . Hard trust requires that participants' identities/credentials are provided in their data spaces and collaboration with those data spaces and that the trustworthiness of the interoperability solution is adequate for participating data spaces and their participants. There must be agreements between the interoperability solution provider(s) and data spaces and between the data spaces so that the existence of agreements can be verified. Hard trust is complemented by soft trust that is based on risk assessment and supports the need for the user to “accept vulnerability based upon positive expectations of the intentions or behaviour of another” [50].
• Data sovereignty, defined in data spaces [27], must also be respected in inter-data space use cases. This means data sharing happens directly between participants, and the data sharing agreement between participants is used as a baseline for data transactions. Trusting possible intermediaries in data processing and transfer, and legal and governance guidelines must be respected too.
• Use of the interoperability solution with different types of data space technologies must be supported. Data spaces can be implemented in multiple ways, the use cases, technologies adopted, and business interests will lead to various non-compatible solutions. This leads to parallel implementations in data spaces in core services such as identity management, publishing and discovery, data sharing contract management, and actual data transactions. A positive consequence is the reduced complexity of structure and management with increasing number of collaborating data spaces.
• Separation of concerns and functionalities so that a modular and easily manageable implementation can be created. The autonomous and self-standing software modules simplify the deployment and maintenance. It also gives companies more freedom to configure their systems according to their needs. Naturally, communications between the functionalities need to be handled, and it must be possible to build working integrations between the functionalities and user systems.
• Support the user in the complex tasks needed in the data transaction. Sharing data requires trust that can be achieved only by understanding the risks associated. The number of data providers and consumers will grow, setting different requirements for publishing, searching and transferring data. Cross-sectoral use cases combine actors from various cultures and practices that need support for collaboration both on human and technical levels.

### 4.2 Functional perspective

**Fig. 5:**
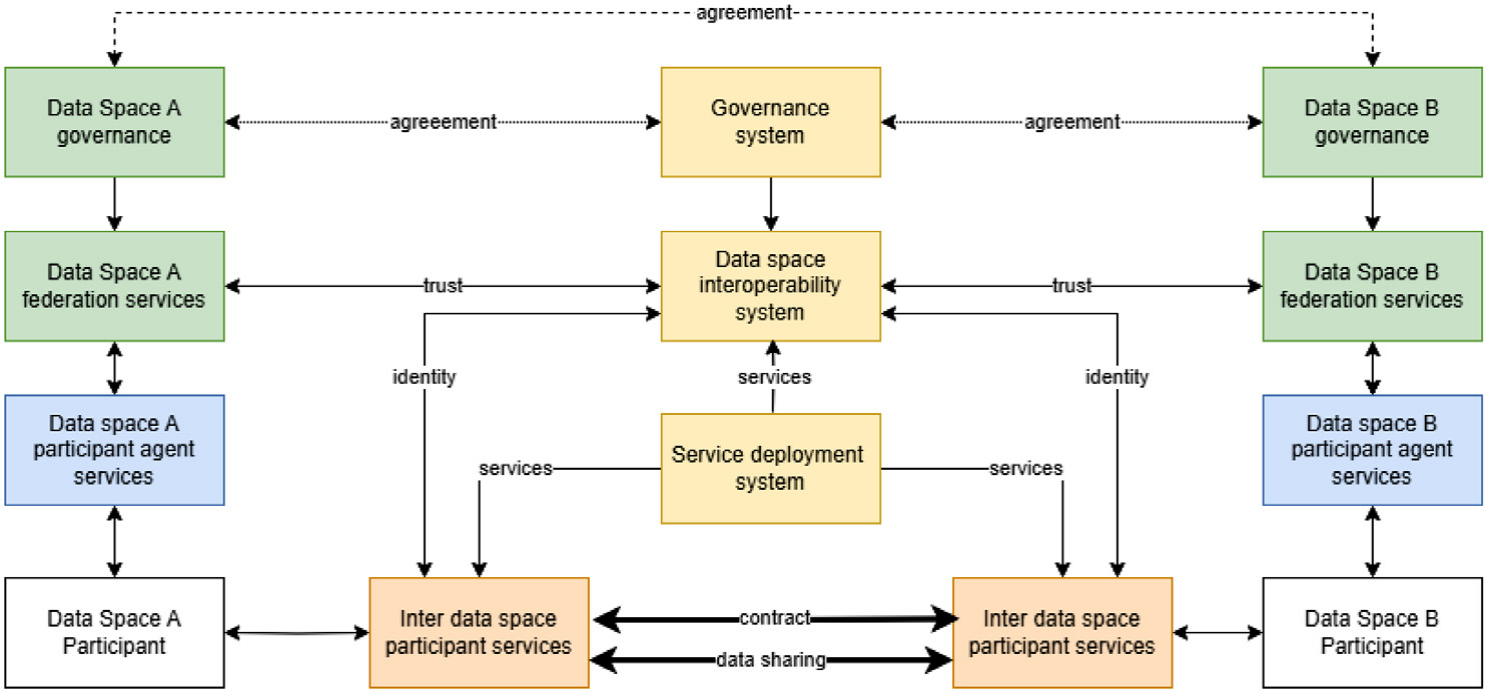
Overview of functional perspective of data space interoperability architecture (DSIA).

The functional perspective of the data space interoperability approach is to have systems and services between data spaces (green/blue boxes) that provide the capabilities for contract-based data sharing across data spaces (Fig. 5). The approach consists of three main subsystems (yellow and brown boxes): The governance system, the data space interoperability system, and the deployment system.

The **governance system** is responsible for creating a legal and trustworthy operational environment for data spaces, governance authorities, and the participants in those data spaces. The aim is not to create an additional governance framework but to implement the collaboration agreements that enable inter-data space data sharing.

**The data space interoperability system** supports cross-data-space trust, identity and access management, visibility of data product offers, and access to participant information. It also includes **inter-data space participant services** providing common solutions for data sharing, contract negotiation, execution, and data transfer so that problems related to possible technical differences in data spaces can be avoided. The participant services can also include services for all phases of contract-based data transactions, such as risk analysis, search support, data transformation, data applications, etc.

As the name suggests, the **service deployment system** provides services for deploying components to participant systems. The deployment system includes support for participant service deployment, an application marketplace and the implementation of an identity provisioning system for DSIA components. Application deployment, based on a containerised runtime environment for applications and services, provides a convenient away to implement this.

### 4.3 Ecosystem perspective

**Fig. 6:**
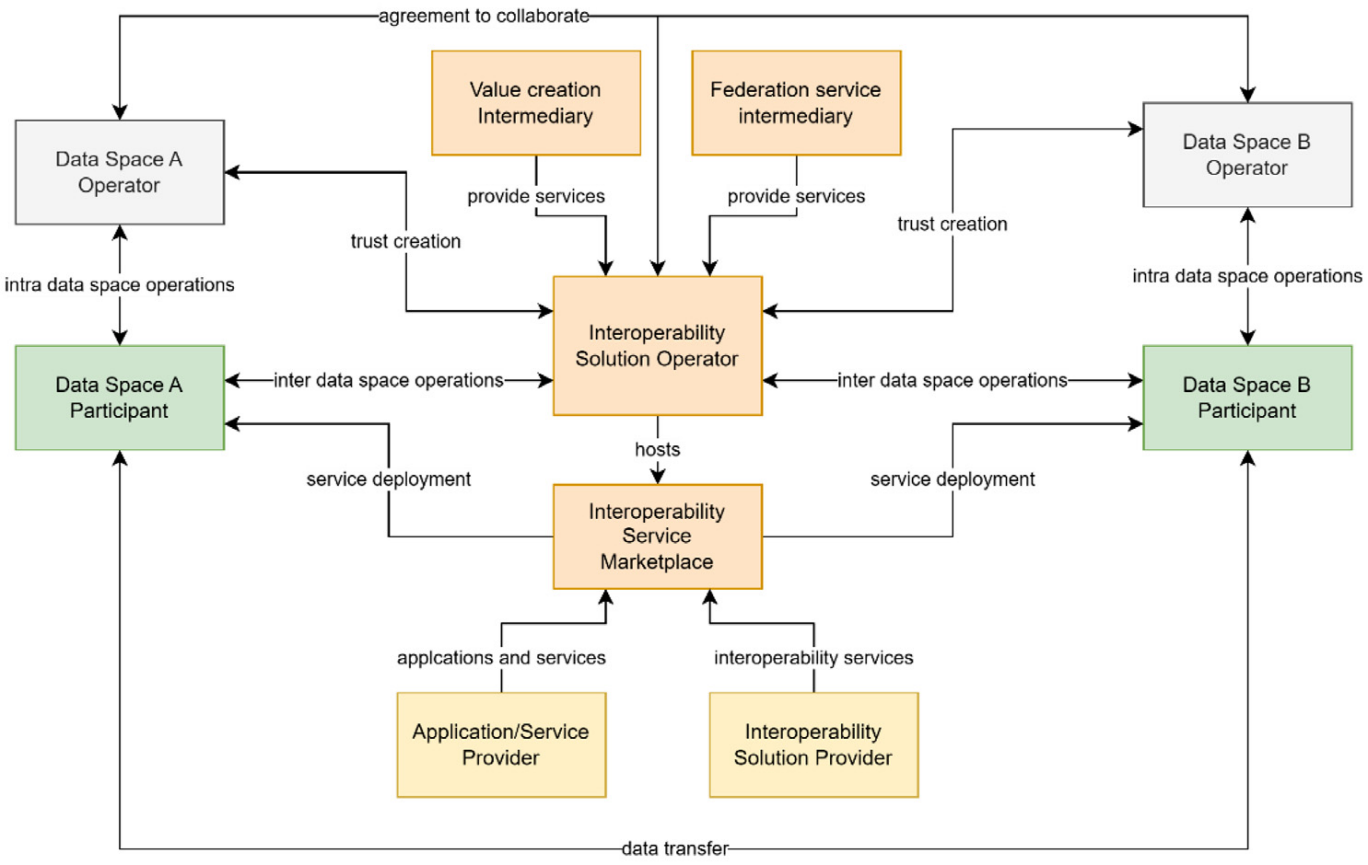
Ecosystem diagram. Actors related to DSIA technology providers are in yellow, actor related DSIA service provisioning in orange.

The business ecosystem of DSIA is depicted in Fig. 6. The main idea is an **interoperability solution operator (DSIA Operator)** between data spaces acting as an intermediary. It gets the technical implementations of services from **the interoperability solution provider,** which develops the software implementations and integrates different applications from **application providers**. The scope and purpose of the application are not limited; the potential needs of users define it. The architecture allows both stand-alone applications and applications deployed as participant services. To support the deployment of its solutions, the interoperability solution operator may host or collaborate with an **interoperability service marketplace** that is a marketplace for DS2 applications.

Data spaces are based on trustworthiness, and core principles in their implementation are known identities of service users, trustworthy services, and common operation rules. All the participants are committed to these principles. To maintain this trust, the **data space operators** must agree that collaboration is allowed and supported by such a DSIA operator and the participants. This agreement is called **a collaboration agreement**. It is a three-party agreement that states the basic collaboration rules, data spaces and participants' obligations, and the DSIA operator's obligations. When the agreement is in force, the **data space participant** can join the interoperability solution, get access rights and install or access the needed services. The choice to join an interoperability solution is made solely by participants and where the collaboration agreement only enables it.

In the implementation of the services of a DSIA solution, the DSIA operator can use either **federation service intermediaries** and/or **value creation service intermediaries**, who can operate respective services. All the intermediaries must agree to follow the operation principles of the DSIA solution.

### 4.4 Data models

Data models in this section describe the contents of different data objects needed in interoperability solutions. The data objects related to offerings and data-sharing contracts are essentially the same as in data spaces. The collaboration agreements between data spaces and a DSIA operator replace the rulebook of data spaces. The participant agreements are not needed as the trust in participants is already created when they have joined the data spaces, and interoperability solutions trust that.

#### 4.4.1 Collaboration agreements between data spaces and operator

A collaboration agreement is needed for pairs of data spaces, whose participants want to exchange data, and the DSIA operator. A single agreement can cover multiple data space collaborations if formulated to cover each data space pair collaboration. The collaboration agreement defines the requirements for data spaces themselves and the DSIA operator.

The collaboration agreement does not need to be machine-readable, however structural representations obviously allow more efficient and automated process. Regardless, only generic descriptions of needed elements are given. The mandatory elements of the collaboration agreement are:

• Identification of data spaces.
• Description of identity validation services of data spaces so that DSIA operators can validate the identities of data space participants when using DSIA federation services.
• Rules of collaboration between data spaces to ensure the compliance of inter-data space activities with the rules of each data space involved. The degree of detail in the compliance depends on the data spaces themselves, the purpose of collaboration with another data space, and the DSIA operator's operation rules.

If data spaces have rules or policies that need to be implemented in data-sharing agreements, they must agree on the likely subset of rules that must be applied in inter-data space sharing. When a data space has compulsory rules, they also need to provide policy enforcement services, and these services should also be made accessible for inter-data space operations.

#### 4.4.2 Offerings

The purpose of data products is to make data into manageable assets for business transactions. As the implementation of offerings may vary, the uniform data product model must be used in the case of inter-data space solutions. This may require participants to modify or create new data products for inter-data space data sharing.

The data product consists of two main parts: resources and offerings. The resources are:

• Data asset: A collection of bits in some format with some purpose and value for the user.
• Data service: A software service used to execute the transfer of data assets from data providers' data storage. Data service can be a database API or web server, for example.

The offering is the description of the data product, and there can be multiple offerings for each data product, for example, for different purposes or target customers. The offering should contain all the information that potential data consumers need to assess the suitability of the data product for its purposes.

The basic contents of the offering are:

• Description of data asset, i.e., what the data is, its format, and its technical characteristics.
• Description of data service, i.e., the service type and the service API description. The user must understand how to access the data. This description does not reveal the address of the data service.
• Data provider information, i.e., the self-description of the data provider. The consumer needs to be able to assess the trustworthiness of the data provider.
• Initial data-sharing contract, which will be used as a basis for contract negotiations. The consumer needs to know the usage rights for data, the evidence of the provenance data, pricing information, access options, technical characteristics important for the contract, etc. Details are described in the next chapter.

Other information may be anything that the DSIA operator wants to add to data products. It can also include user-definable parts so that data providers can add information they feel is needed.

In the context of data products in inter-data space scenarios, a new requirement is related to the visibility of offers. Data providers must be able to define who can see their offerings, so new metadata on visibility across data spaces must be defined.

#### 4.4.3 Data sharing contracts

Data sharing contract is an outcome of contract negotiations. The inter-data space data sharing requires a common data sharing contract approach. Common contracts can be achieved through transformations from data space-specific contracts, but in the DSIA architecture, an Eclipse Data Space connector-based contract model has been chosen.

The contract has two purposes. Firstly, a data‑sharing contract is a legal agreement between the data provider and the data consumer. It defines what data is exchanged and how the exchange takes place. It also specifies contract conditions, permitted data usage, and how exceptions are handled. In this sense, it is like any legal agreement between legal entities. Secondly, a data-sharing contract is a (ideally) machine-readable set of instructions used for trustworthy data transactions in the data-sharing system. During the data transaction, those instructions (also called policies and conditions) that can be executed with a data-sharing system are executed and evaluated. For parts that are not machine executable, the implementation is transferred to external and possibly non-software processes, and the outcomes are fed to the data system using the system's user interfaces. The process continues until the transaction ends, and the contract can be terminated.

**Fig. 7:**
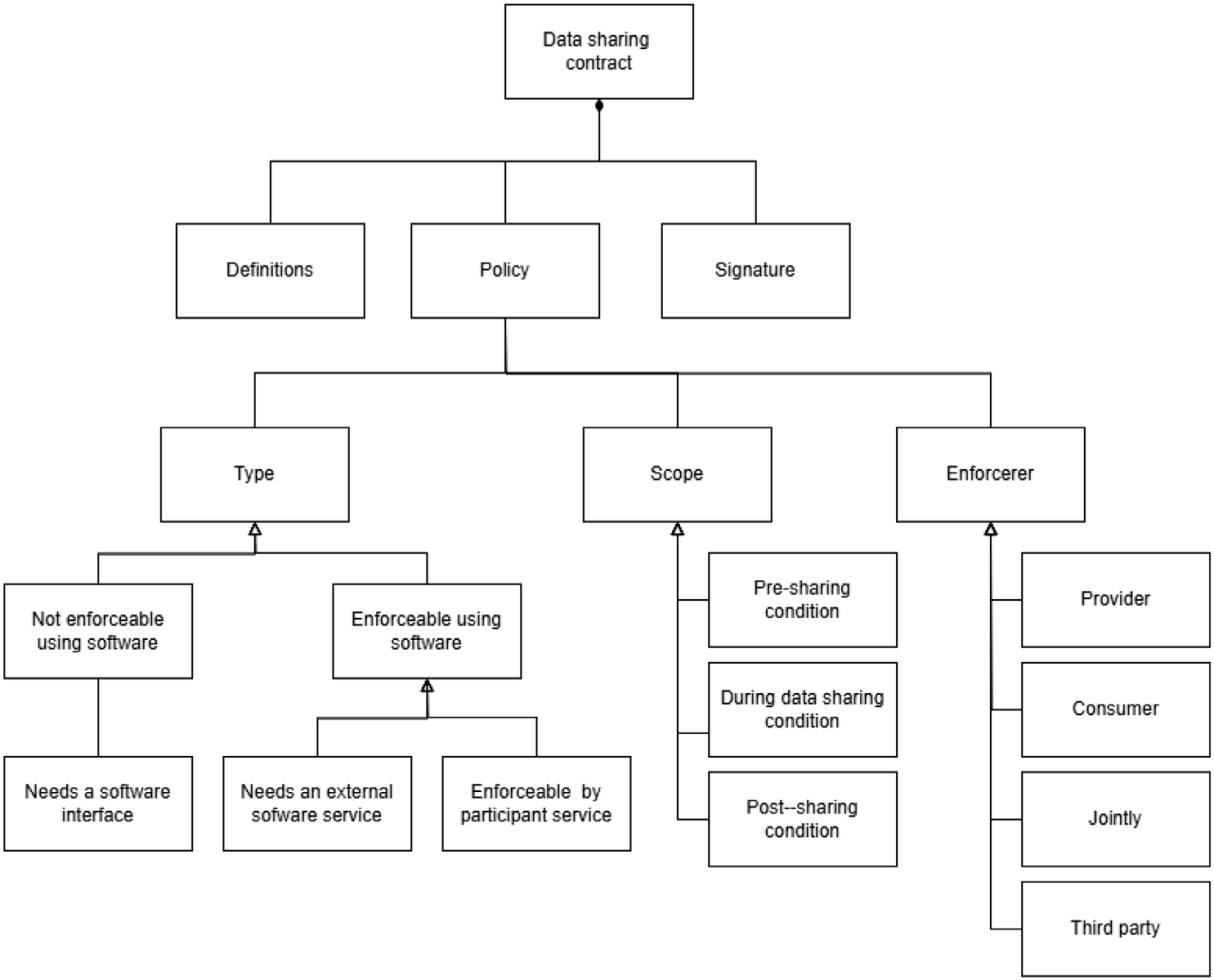
Data sharing contract.

The data-sharing contract can be modelled using the Open Digital Rights Language (ODRL) [51]. The basic structure is depicted in Fig. 7. The main elements are definitions, policies, and signatures. The policy part is meant to be machine-readable. Policies: 1) need to define the type of policy, i.e. how it can be enforced; 2) what is the scope of the policy, i.e., does it needs to be validated before the data transaction during the data transaction or after the transaction, and 3) who is the enforcer of the policy, i.e., is it provider, consumer, operator, or some third party and how the validation service can be accessed.

### 4.5 Functional system architectures

#### 4.5.1 Interoperability governance system

**Fig. 8:**
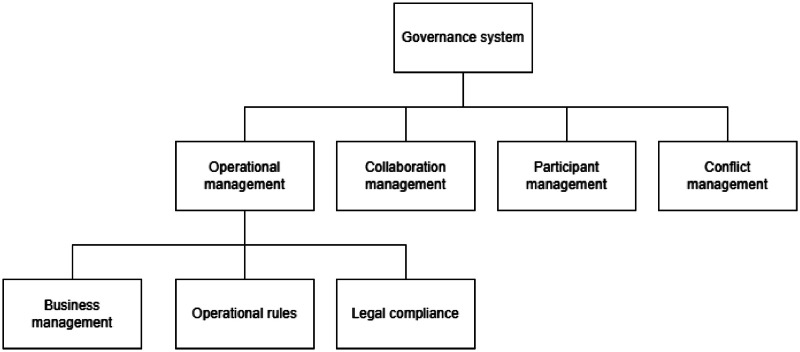
Structure of the DSIA governance system

The governance system model is based on the governance principles defined in DSSC Blueprint [27], except that the interoperability solution can rely on the governance principles of data space that are decided to be used. The structure presented in Fig. 8 is close to a normal data space model.

The governance system consists of operational management, collaboration management, participant management, and conflict management. The system is implemented with human processes, but it activates other systems, such as when new collaboration agreements are signed, or new participants join a DSIA system.

**Operational management** depends on the operator's interest in how to manage the DSIA solutions. It includes business management, definition of operational rules, and legal compliance issues. Business management implements the business processes between DSIA operator, service intermediaries, data space governance authorities, and participants. The implementation depends on a chosen model, such as participation and transaction fees. Operational rules should define and document all the processes used in the DSIA solution. Legal compliance is mandatory for operational principles, all agreements, and data transactions. Responsibility is divided among stakeholders.

**Collaboration management** deals with collaboration agreements between data spaces and a DSIA operator. A DSIA operator oversees the agreement process, and when everyone has signed the agreements, the governance system informs the data space interoperability system that identities should be given to data spaces and collaborating data space pairs. It also leads to adding data spaces to the data space participant registry hosted by the DSIA interoperability system. It also needs to ensure that data spaces enable the identity validation of their participants to the DSIA. The data spaces that want to use a DSIA solution need to be controlled by the DSIA operator so that no unwanted data spaces can join and reduce the trustworthiness of DSIA.

**Participant management** controls the joining, leaving and monitoring of participants who want to use the federation services of DSIA. During the joining, the identity is added to the registry hosted by the interoperability system. Checking participant identity via its data space is enough for evaluation as trust is based on the participant’s role as a data space member. After acceptance, the participant is given the right to deploy participant agent services from the service deployment system. When the legal entity leaves, the participants' identities and services are removed from the registry. There can be other types of participants such as technology providers, data space operators/authorities, and even apps themselves that can be managed with similar process. The DSIA operator may define the monitoring of participant behaviour. The current architecture does not propose any automatic monitoring services. However, it is possible and likely that in some large-scale operational systems, there will be a need to monitor how participants follow the operational rules and if there are indications of malicious behaviour.

**Conflict management** includes conflict reporting and resolution processes. Participants can report problems with their data-sharing contracts or other functions in the DSIA system. The DSIA operator can suspend and release the contract execution processes under conditions defined in operational rules. It is up to the DSIA operator and data-sharing contracts to define how conflicts are resolved, but this may also include legal processes.

#### 4.5.2 Data space interoperability system

A data space interoperability system enables data sharing across data spaces. It consists of three subsystems. 1) The data sharing subsystem is the actual transfer of bits between data space participants which is the core of data space's interoperability. 2) The data pipeline subsystem enables the planning and control of data pipelines consisting of different functionalities that simplify the creation and use of data products. 3) The support subsystem comprises applications and services that help users manage the complexities and problems of expanding the data ecosystem. In this context, this paper focuses on understanding the risks, simplifying user interaction with intelligent chatbots that assist in search and configuration tasks, and delivering data to be used through data spaces.

### Data sharing across data spaces

**Fig. 9:**
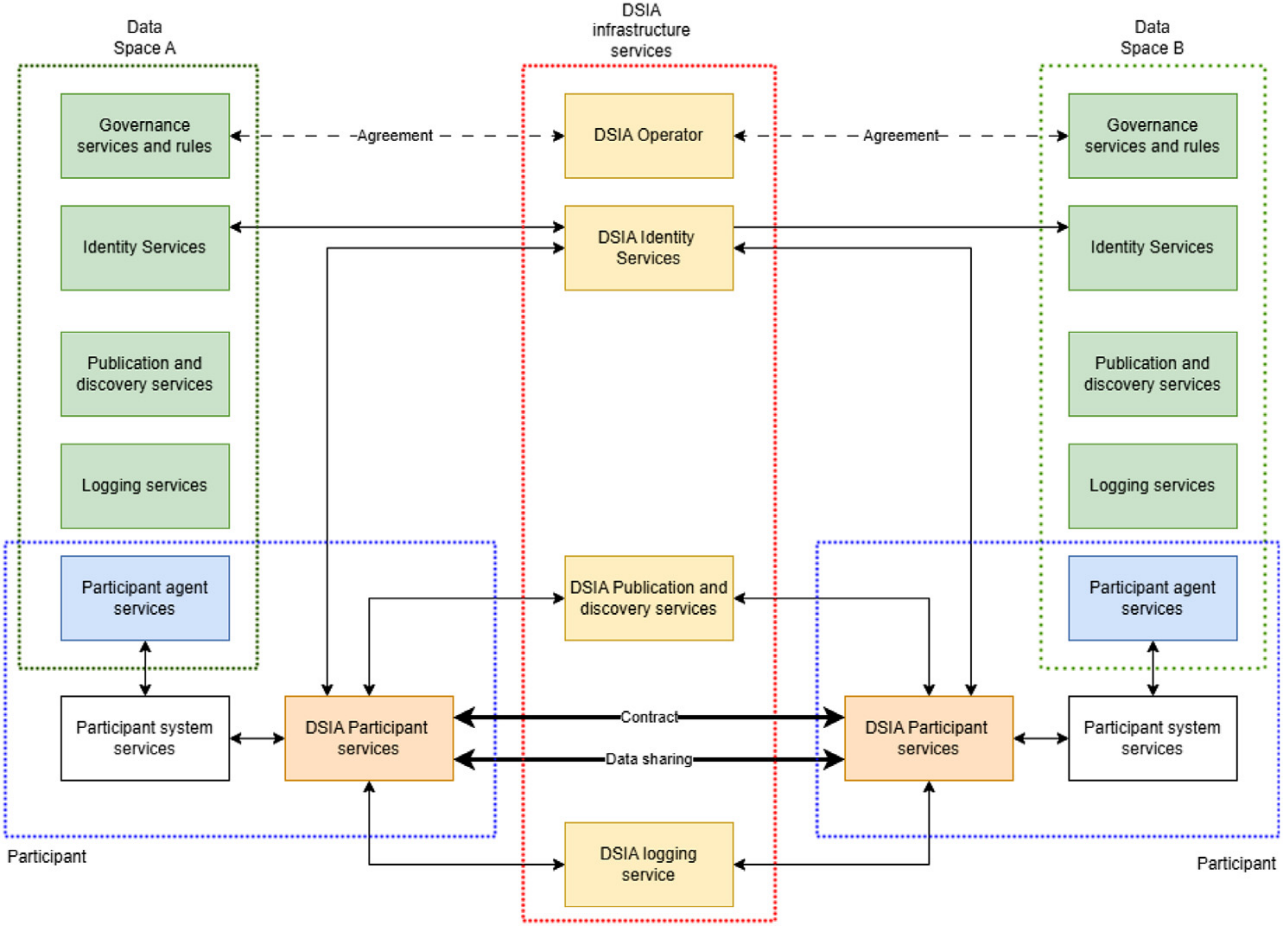
Overview of inter-data space data sharing architecture of DSIA.

The core of DSIA data sharing is the capability to transfer bits securely and trustworthy through data space boundaries. Fig. 9 presents the main components of the DSIA interoperability system.

The solution's core is to use a common **DSIA Connector** and the current DSIA concept is to utilise the commonly implemented Eclipse Data Space Connector with suitable extensions. From the participant's perspective, the DSIA Connector is parallel to the participants' own data space connector(s). Due to the problem of having different connector technologies in participating data spaces this is the only possibility of solving this unless (or until) connector specifications are globally standardised. The DSIA connector solves the issues related to P2P communication, i.e., contract negotiations, execution, and data transfer, that can all be implemented using data space protocol [45]. Current extensions foreseen are related to policy management, data plane, search and discovery, and user interfaces, and the motivation is mainly to improve the usability of the Eclipse connector, extending with inter-data space features, instead of creating new features.

The big challenge in inter-data space operations is the trust framework, as they have been designed to support intra-data space operations only and modifying them is a complex option. **DSIA identity services** solve the problem by providing:

• Identity provisioning for data spaces, data space pairs, DSIA services, and DSIA connectors.
• Identity validation services for DSIA services and components.
• Identity validation for participant identities in collaboration with source data space identity management.

The DSIA identity service has a data space collaboration registry for collaboration identities and a DSIA component identity register for DSIA component identities. The participant identities are checked from the participant data spaces’ identity services by DSIA identity management.

The process is:

1. When the DSIA connector or DSIA service receives a message, the message contains the sender's and sending components' identities.
2. The receiving entity sends the incoming message with its own identities to the DSIA identity validation.
3. The DSIA identity validator checks whether the receiver and sender are members of the data spaces that have agreed to use DSIA and that the collaboration agreement between these two is OK.
4. Then it checks that both receiver and sender DSIA connector identities are valid.
5. It checks from the receiver's and sender's own data spaces' identity validation services that these legal entities still have valid identities in their own data spaces.
6. If all checks are OK, the message can be trusted.

**DSIA publication and discovery service** hosts data offerings and participant information published for inter-data space collaborations and executes search queries from DSIA participants. The interface to DSIA publication and discovery service is data space catalogue protocol, and all requests come through DSIA connectors. The processes for data offering publication and search are similar to those where all participants are in the same data space. The key difference between the IDSA metadata broker component is that the visibility control must implement the inter-data space visibility rules defined in offerings and for participants' information.

**DSIA logging service** is similar to the IDSA clearing house. It logs the data-sharing contract event to a persistent store that is accessible to the contract parties, the DSIA operator, and legal authorities in specific conflict situations. Other events and processes can also be logged here dependent on data space and participants’ needs

From a user perspective, the DSIA approach is a lightweight version of a data space. After a data space has accepted DSIA collaboration, joining DSIA is a simple process for a participant. More challenges come with the preparation of data products. The original home data space may have different ways of implementing everything, which causes additional work for the partner. In the worst case, it may mean duplication of data services, offering descriptions, and contracts. In the best case, the same models and services can be used in both.

### Data pipelines across data spaces

Data sharing between companies requires common protocols and data formats. However, sharing data across data spaces and sectors increases diversity in ontologies, languages, formats, and quality levels. This diversity increases the risks related to data interoperability. The data space and its contract-based data transaction concept require that the data and data service properties are explicitly defined, making it difficult to provide data in multiple formats. The DSIA supports having a data pipeline between the data transaction services and the participant system. The idea is to enable data transformations and services such as quality validation, data curation, etc., as a part of data transactions.

The data pipeline can exist at the data provider, data consumer, or both. It can be external to data space services or integrated into contract execution as a policy enforcement function. The external pipeline is fully controlled, and the responsibility is with the legal entity that hosts the pipeline. The Integrated pipeline operates during the data transaction under the control of the DSIA connector, and the pipeline configuration must be included in the data-sharing contract as one contract execution policy. It is up to the DSIA operator to decide which approach is used.

**Fig. 10:**
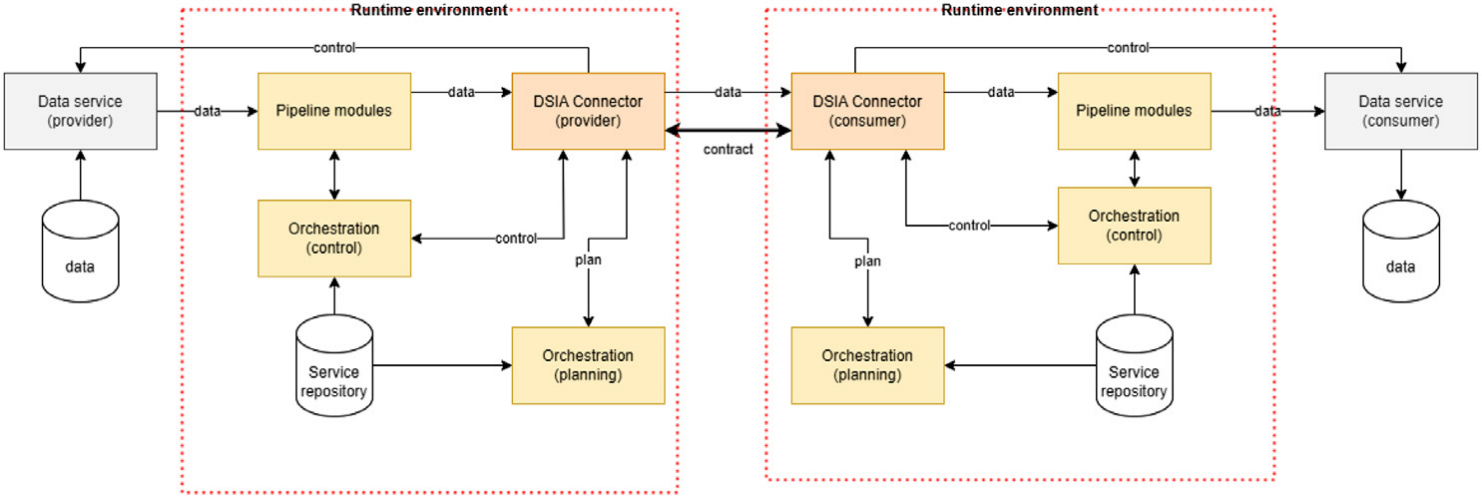
Data pipeline overview.

The main components of the data pipeline are presented in Fig. 10. **The pipeline orchestrator** has two functionalities: a pipeline **designer** and a pipeline **runtime controller**. The designer is used to plan the pipeline workflow using the pipeline applications between the data service of the owner and the data plane of its DSIA connector. The outcome of the planner is **a pipeline workflow plan**. The designer is used before the data transaction starts, and in the case of an integrated pipeline, the plan must be added to the data-sharing contract. The runtime controller implements the plan either based on user command or instruction from the policy engine of the DSIA connector. **Pipeline applications** are DSIA operator-approved services that execute data operations for the data asset being transferred. The DSIA approval includes defining the application interfaces so that the orchestration planner can enable the interoperability of connected applications or interfaces. **An application repository** is a storage of applications possessed by the pipeline owner. Applications are acquired from the DSIA operator's or intermediary's portal's service marketplace.

The data pipeline and the portal implement the app store concept of IDSA in a more loosely coupled way than described in IDSA. If the applications are validated and certified by the DSIA operator, and the marketplaces are validated, this approach could be considered as trustworthy as IDSA.

### Data space supporting services

Data spaces and inter-data space data sharing are extensions of an organisation's data management system as they provide means to get/provide data from/to external organisations via contract-based data transactions. This introduces two challenges: 1) how to integrate the data spaces and interoperability solutions to existing data management solutions, and 2) what kind of additional support is needed for an organisation in operation with possibly previously unknown parties from different domains, practices, and cultures.

Current data space solutions provide little or no support for user interaction and processes. Typically, they provide API descriptions and user interfaces (UI) for technical people, but the actual integration and end-user support are missing. As data management implementations vary depending on an organisations' domain and scale, it is impossible to define in detail how the integration should be performed and how the services described here actually divide into data management or interoperability solutions. In DSIA, this is addressed with the concept of supporting services and mapping the services to the contract-based data transaction process. The spectrum of choices in the actual services is infinite, and giving a complete list is not in the scope of this paper. In the following section, some examples of what kind of support services could be useful are given, and section 0 describes a set of service implementations.

The first phase in a contract-based data transaction is **creating and preparing data**. As data is assumed to have business value, it is essential to keep it confidential and to make it useable, potentially sellable, without revealing its content. Confidentiality requires keeping the whole process of data creation and collection in a **secure environment**. Making data useable means **setting up a data service** for the data asset that controls access to the data and connects the data service to the DSIA connector. It also involves the **creation of a data offering** that is used to inform potential data consumers what is offered and how it can be accessed when a contract exists. Both setting up a data service and creating a data offering are processes where advanced AI tools could provide support by automation.

The second phase is the **publication and discovery of data offerings**. When the size of data spaces and possible interoperable data spaces increases, the number of data offerings increases and the complexity of search increases. **Formulating exact search queries** will thus be more important. Equally, **filtering and understanding search results** will be challenging as they may be context-dependent, have different languages, use different ontologies, and have different semantic interpretations.

**Assessment of the feasibility** of the data transaction and **the decision to open the contract process** are the next phases after the identification of the data product. Understanding the risks associated with the sharing of confidential data is needed. This involves analysis of the trustworthiness of the parties involved, their systems, security threats involved in data transfer, and other identifiable risks. Data offerings and participant information inside data spaces can be used, but external information sources should also be considered in any business decision. Legal compliance of planned data transactions needs also to be verified. The amount of effort to be put in depends naturally on the data product's value and the parties' familiarity.

**Contract management** consists of **negotiation, initialisation, execution**, and **termination** processes. The main objective of data spaces is to automate these as much as possible. However, especially during the contract negotiation process, human decision-makers may be required to be connected to the loop. The data-sharing contracts may be very simple technical descriptions of data transaction processes that are accepted as such or with few changes in transfer parameters. The other extreme may involve complex data processing, policies that can only be enforced using external services or even human processes, and the participation of both provider and consumer.

The basic supporting services in the negotiation phase is the transformation of machine-understandable policies into the human-understandable format and vice versa so that human decision-makers can evaluate and create policy proposals.

In case the data services and data product representations between provider and consumer do not match, there is needed to plan and instantiate a suitable set of data transformations, data processing, and service configurations or adaptions as a data pipeline, as configuration of DSIA connector's data plane, or as configuration of data services. Deciding what services to use, how they can be connected, etc., can be a complex process where AI-assisted tools might be helpful.

During contract execution, the implementations of external policy enforcement functions provide plenty of room for supporting functions ranging from data quality validation and interaction with human processes to payment systems. Due to data sovereignty requirements, the data provider should also have the possibility to control the execution of data transactions, and both parties should have the means to verify that the contract has been executed correctly. The DSIA interoperability system provides logging services, but the analysis and interpretation of logs could benefit from supporting AI tools.

Data-sharing contracts can limit the **use of data** to a specified purpose. This can be implemented as a data analysis service within the DSIA runtime environment that can be considered trustworthy if the DSIA operator has implemented adequate security and trustworthiness mechanisms for it and its services. If the data-sharing contract allows the transfer of data outside the DSIA connector, the data providers' capabilities to monitor the use of data are limited unless additional monitoring services are explicitly defined and instantiated. Another option is the detection of misuse of data and getting the protection of data rights holder's rights from the legal system.

#### 4.5.3 Service deployment system

**Fig. 11:**
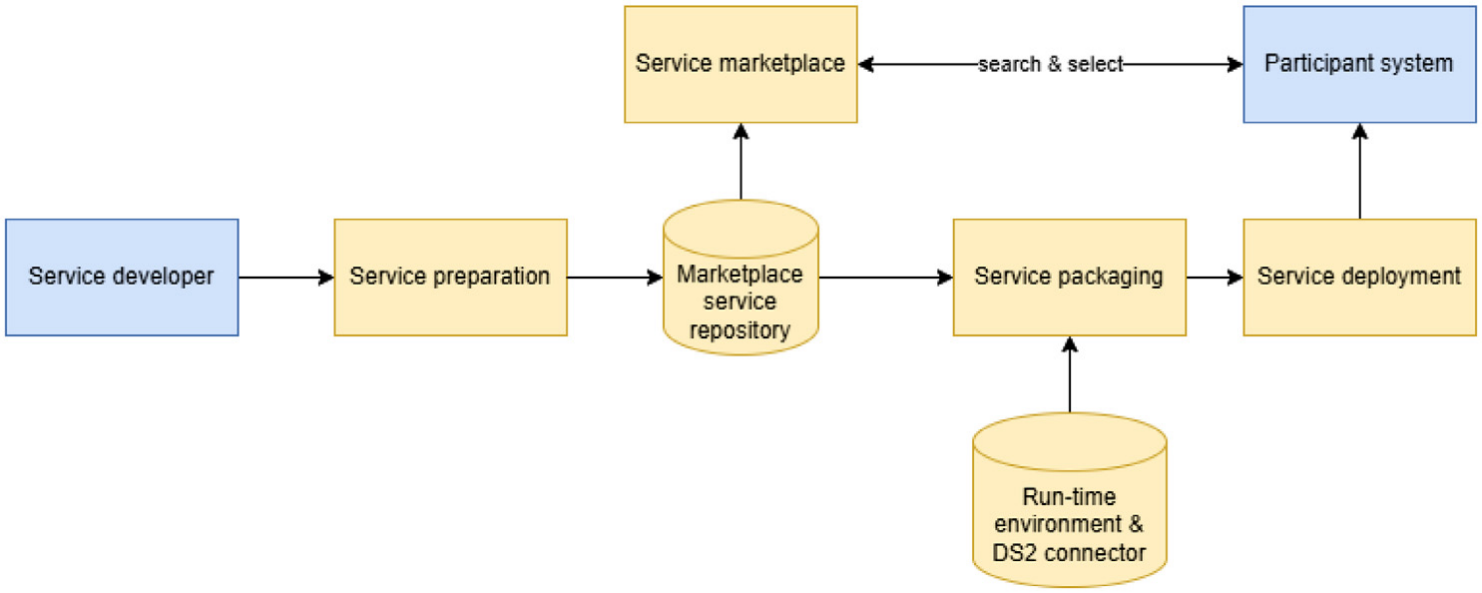
DSIA deployment architecture

The objective of the DSIA service deployment system (Fig. 11) is to provide a DSIA service marketplace that allows DSIA participants, intermediaries, and operators to install a runtime environment based on containerisation and a set of DSIA services and DSIA connectors as containerised applications or services into their systems/servers. The service deployment system also supports service and application developers in publishing their solutions in the service repository of the marketplace as applications that can be deployed into the DSIA runtime environment.

The key principle of the service deployment system is that all the services are as stand-alone as possible so that the benefits of containerisation can be fully exploited. The goal of its runtime environment is to be as independent as possible from the user's system.

#### 4.5.4 Validation of DSIA

The proposed DSIA can be validated by implementing its core features and evaluating how well they serve the needs of possible use cases. The challenge is the huge diversity of possible data spaces and use cases making it impossible to provide complete assessments. The main implementation characteristics that need to be considered are solutions ability to serve different types of data spaces and how to execute the contract-based data transaction across data spaces. The DSIA proposes to have dedicated participant services that solves the technical communication level interoperability issues, but there are open issues due to variety of data and trust issues related to operational rules of data spaces such as following:

• Making and verifying agreements between the data spaces about the collaboration.
• Verifying the status of data space membership of interoperability participants across data space boundaries.
• Supporting the data sharing contracts and policy enforcement across data space boundaries.
• Providing support for data transactions between various types of data and data services.

All these issues are highly implementation and use case dependent. The instantiation of DSIA must be done based on the data spaces to be connected to it and what kind of data transactions need to be done. The validation can cover only those implemented features. The DSIA allows multiple implementations of the same functionalities, but the DS2 implementation presented in the following chapter has only a small subset of possibilities. In the following chapter it is shown how the four issues above are addressed on DS2 use cases.

## 5 DS2 Implementation of DSIA

**Fig. 12:**
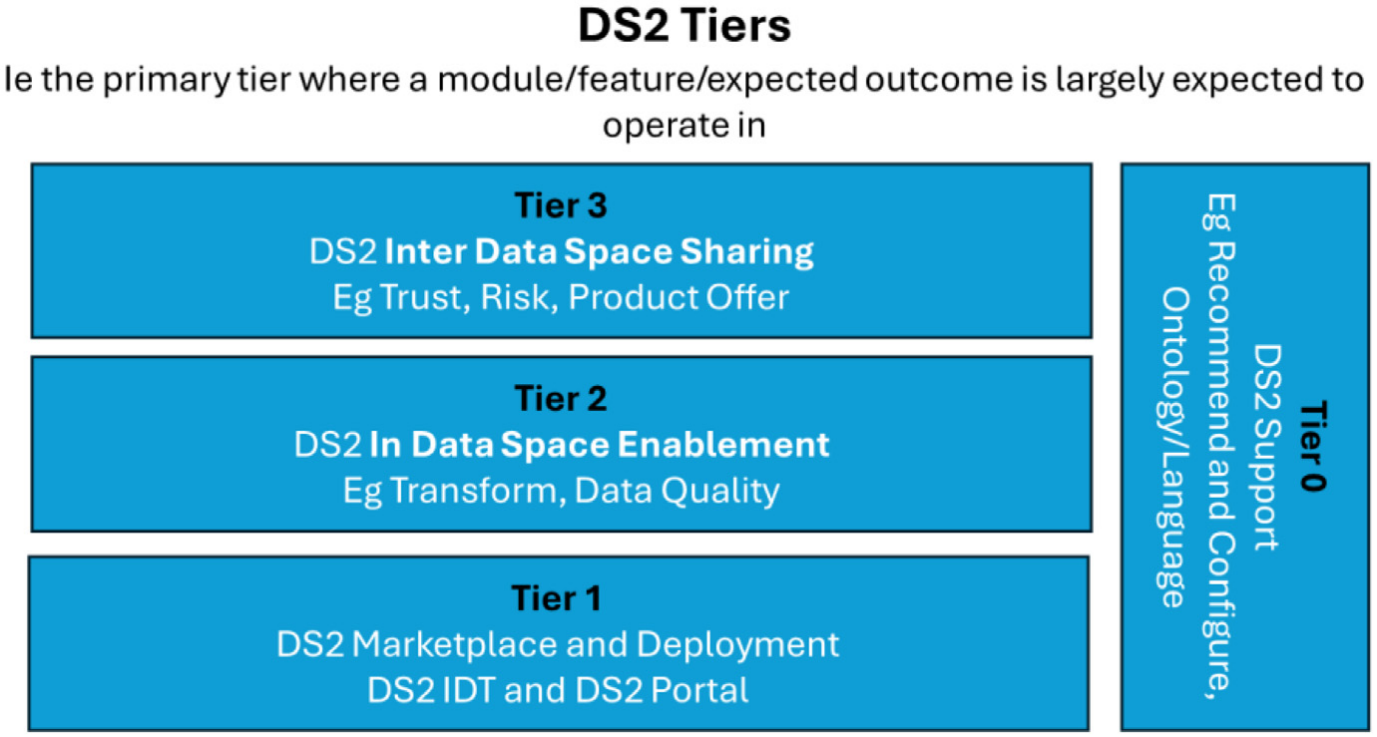
DS2 module tiers.

The DS2 approach^1^ implements the core features of DSIA presented. It is based on the idea of data space interoperability through an intermediary that provides essential DSIA infrastructure services and a service marketplace from which each participating data space member can deploy a run-time environment called Inter-sector Data space Toolkit (IDT), which includes the participant agent services (i.e., DS2 connector solution), and a participant selectable set of DS2 modules that it needs in the creation of functional data sharing capabilities across data spaces. The network of participants' IDT modules and DS2 operator's infrastructure services create a data-sharing environment based on data space principles of participating data spaces. A prerequisite of this network is the agreement between data spaces and DS2 operators to use and share the services needed for collaboration and to trust the participants of participating data spaces. DS2 consists of software modules divided in Tiers as presented in Fig. 12 according to main purpose of the module.

• Tier 0: DS2 support includes examples of supporting applications of DSIA data space supporting services subsystem.
• Tier 1: DS2 marketplace and deployment contains operations needed for the DSIA deployment system.
• Tier 2: Data Space Enablement contains mainly modules for the DSIA data pipeline subsystem and modules for the DSIA supporting services subsystem. The division between Tier 0 and Tier 2 is related to how close the functionality is needed to the actual data transaction.
• Tier 3: Inter Data Space Sharing has modules needed for the DS2 Operator system that implements the DSIA data sharing across the data space part subsystem.

DS2 also includes the DS2 connector, an extended Eclipse Data Space Connector version. Some of the modules in Tier 3 are extensions or replacement functions for Eclipse Connector such as policy enforcement module. It is also important to note that the data plane of Eclipse Data Space Connector must be designed and implemented during the instantiation phase so that it implements the needed features of DS2.

DS2 focuses on creating modular, configurable applications that integrate functionalities, data, and interfaces into easily deployable packages. This approach allows participant systems to meet their needs flexibly. Additionally, it is an open environment for adding new modules that comply with basic DS2 principles.

The DS2 modules are broadly conformant to DSIA. The data sharing part and DSIA deployment system are covered completely, but in other parts, DS2 gives only example implementations as the design space for modules is unlimited. The DS2 data-sharing system communicates using Data space protocol [45], and the modules interacting with the DS2 connector use Eclipse Connector API [53]. Each module has its user interface with common navigation capabilities supported by the IDT module, allowing users to have an easy-to-adopt common user experience. However, they also have APIs to connect modules directly to each other or the user's data management system.

### 5.1 DS2 modules

The details of each module are in [52], where the description consists of description of use, component definition, technical description, interface description, and in case of open source, the code.

### Tier 3 Inter-data space sharing modules:

• The **Sovereignty Decision Support Module** (SDS) is a risk modelling and assessment tool. Contracts and identity management technologies provide a foundation for secure data sharing, but they are not sufficient on their own to establish trust in the process. Further information needs to be shared between provider and consumer to ensure that the decision maker can take a risk-informed decision when sharing data. From a practical point of view this means that a mechanism for sharing and then analyse some information on the infrastructures and data management systems needs to be setup. SDS employs the "soft trust" methodology.
• The **Digital Rights Management Module** (DRM) enhances the management and security of digital asset transactions through a robust blockchain-based Data Rights Management (DRM) system. It is designed to perform critical functions, including the notarization, tracking, and validation of all data rights transactions both within individual Data Spaces and across multiple participating Data Spaces
• The **Policy Agreement and Enforcement Module** (DS2 PAE) ensure compliance with the established policies and regulations governing data exchange among users in different data spaces. Henceforth, policies, regulations, and agreements are synonymous with the term policy. Policies are evaluated at the control plane stage of data sharing within the Connector. The policies serve two main purposes: Access Control and for Usage Control. Access Control determines whether access to data is granted or denied. Usage Control dictates how the data can be used once access is granted.
• The **Catalogue Module** (CAT) is designed to support the exchange of data within and across different data spaces. The main goal of DS2 catalogue is to enhance the functionalities of catalogue systems within existing reference architectures, enabling them to support both intra-data space and inter-data space operations. This includes defining data models for data product offers, data product offer searches, and interactions with members of other data spaces, thereby fostering collaboration across different data spaces.
• The **DS2 Identity Module** (IDM) provides a framework for the creation and validation of identities for inter-Data space activities based on the existence of existing data space and their own individually selected identity authorities. This is linked to the IDT module and DS2 connector and allows for a federated approach of the connectors whilst relieving participants from connector interoperability, outside data space change and maintenance issues, and minimising or eliminating the changes to their existing environment.

### Tier 2 In data space enablement modules:

• The **Orchestration Module** (ORC) helps to design and then orchestrate at runtime In-Data space, Inter-Data space, internal, and third-party services which facilitate common data-orientated operations such as transformation of data, checks on data, data updates etc. The orchestrator contains a flexible GUI to design workflows and decision points on these services and run time component to implement the workflow.
• The **Data Retrieval Module** (RET) simplify the effort required to access a data source by automatically generating the required REST call for the Data Service and connector, based on a textual request in natural language.
• The **Data Detection and Transformation Module** (DDT) can analyse and transform data on the fly during the data transaction.
• The **Model Development Toolkit Module** (MDT) provides a set of tools to allow the users to develop algorithms based on the CRISP-DM standard to assist in the whole development cycle (training, test, etc.) and package the algorithms which can be deployed as executable software component.
• The **Data Curation Module** (CUR) Module is invoked on two or more data sets and aims to identify through machine learn data transformations which need to be performed on fields to allow interoperability between the data sets – for example, the conversion of time-data formatting. The required transformation(s) will be automatically selected from a transformation library, and a processing pipeline will be created and executed to curate the data.
• The **Discovery, Assessment, Recommend and Configure Module** (DARC) inquires, discovers and assess though the conversational UI of an AI-driven agent, the capabilities and limitations of DS2 Data Spaces as well as DS2 Modules which will compose the “ideal use” scenarios/ paths that will fit the needs of the end-users. Then recommending to end-users, the best DS2 Modules for the implementation of the selected “ideal use” scenario/ path that is configured to DS2 pipeline.
• The **Edge-to-Cloud Module** (E2C) is used to establish a secure edge-to-cloud connectivity for data providers to cloud-based IoT platforms like Azure IoT or Cumulocity IoT or AWS IoT via a MQTT bridge. The data providers will decide which data to share and with whom. In addition, the data quality can be monitored as well.
• The **Data Inspector Module** (DINS) facilitates the configuration and deployment of processes for real-time data analysis, ensuring data quality and compliance with thresholds set by the parties involved. It performs several key functions: generating notifications based on the values of the exchanged data, executing reactions such as sending requests and notifications to external tools, and integrating with models developed to enhance its capabilities. It is a complement to the Data Share Controller which focuses on control information with both Modules using the Data Interceptor.
• The **Data Share Controller Module** (DSHARE) provides a user orientated view of control plane information related to a specific exchange of data to monitor its status and to potentially limit or block it. It will access data through a Data Interceptor component which it shares with the DS2 Data Inspection component (DINS) which operates more at the data level. It can be seen an In-Data space enablement Module. Its role is especially important in an Inter-DS environment to provide extra monitoring and control of the data exchanges when partners are less known.

### Tier 1 Marketplace and deployment modules:

• The **Data Marketplace Module** (DMK) will provide a marketplace for data and data models. It will allow the registration of data from a catalogue, record all transactions, and communicate transactions to any external system if required (e.g. Data Rights Management Module, Clearing House). Data will not be stored in the Data Marketplace Module. It will support both datasets and algorithms.
• The **Inter-sector Data space Toolkit** (IDT) is the core enabler of DS2 who purpose is to be deployed in front of participants data source/spaces and network connected to any other IDT-enabled data source. As such its aim is to run all DS2 Modules, including the **DS2 Connector,** the core Module for Inter-Data space communication and data transfer, and the Containerisation Module for DS2 Module deployment. IDT contains the core Kubernetes runtime to run all containerised Modules and a series of additional open-source software for Module management.
• The **Portal Module** (PORTAL) provides a user and developer friendly portal allowing data space participants to register and select DS2 Modules which can then be packaged into an IDT environment and then subsequently deployed by participants enabling both In-Data Space and Inter-Data Space operations. As such it includes functionality for developers to include Modules, users to find those Modules, to trigger the packaging through links with the containerisation Module, as well as supporting functionality for data space support, data space resources, registration and identity management, and administration. It also provides support for the Data Marketplace.
• The **Containerisation Module** (CONT) allows easy and automated packaging and deployment of Modules on the IDT Kubernetes runtime subcomponent environment. The containerisation Module leverages on custom Helm Chart descriptors to automatically convert them into full Kubernetes Helm Charts representing the Module, based on standard base templates located in the DS2 Portal Marketplace. The Helm Charts are then deployed on the IDT Module.

### Tier 0 Support modules:

• The **Culture and Language Module** (CLM) aim to give a data consumer a better understanding of what data offers exist both within their own data space and in other data spaces, which may come from different sectors or countries and in different languages. This will increase the potential for use of a data offer. This is achieved through transformation of human language in shared information and queries into a rich, searchable hierarchical ontological description of the offered data set.
• The **Multi-cloud Module (**MCL) enables efficient transfer of discreet data, vast amounts of data, and streaming data between participants of data space from data stores that are distributed across multi-cloud storage infrastructure. MCL includes intelligent data placement and caching at data space provider participants with a data space consumer participant requesting such data and provide services through use case applications(s). It will also ensure data exchange happens over secure connections using the Security Module (SEC).
• The **Security Module** (SEC) covers data security, data protection, and privacy with a focus on securing the edge-to-cloud data enablement and ensuring data quality and privacy. This involves implementing secure communication protocols, robust authentication mechanisms, encryption, anonymisation, and continuous monitoring of events in the DS2 ecosystem. The DS2 architecture is designed to manage large volumes of data, facilitating data-driven decision-making while maintaining stringent data security and data privacy standards.
• The **Policy Creation Module** (PCR) is a tool for creating policy descriptions in ODRL. Tool can be used both for creation of data space policy needed in inter-data space data sharing during data space’ agreement process, or for creation of policies for data sharing contracts.

### 5.2 DS2 deployment scenario

**Fig. 13:**
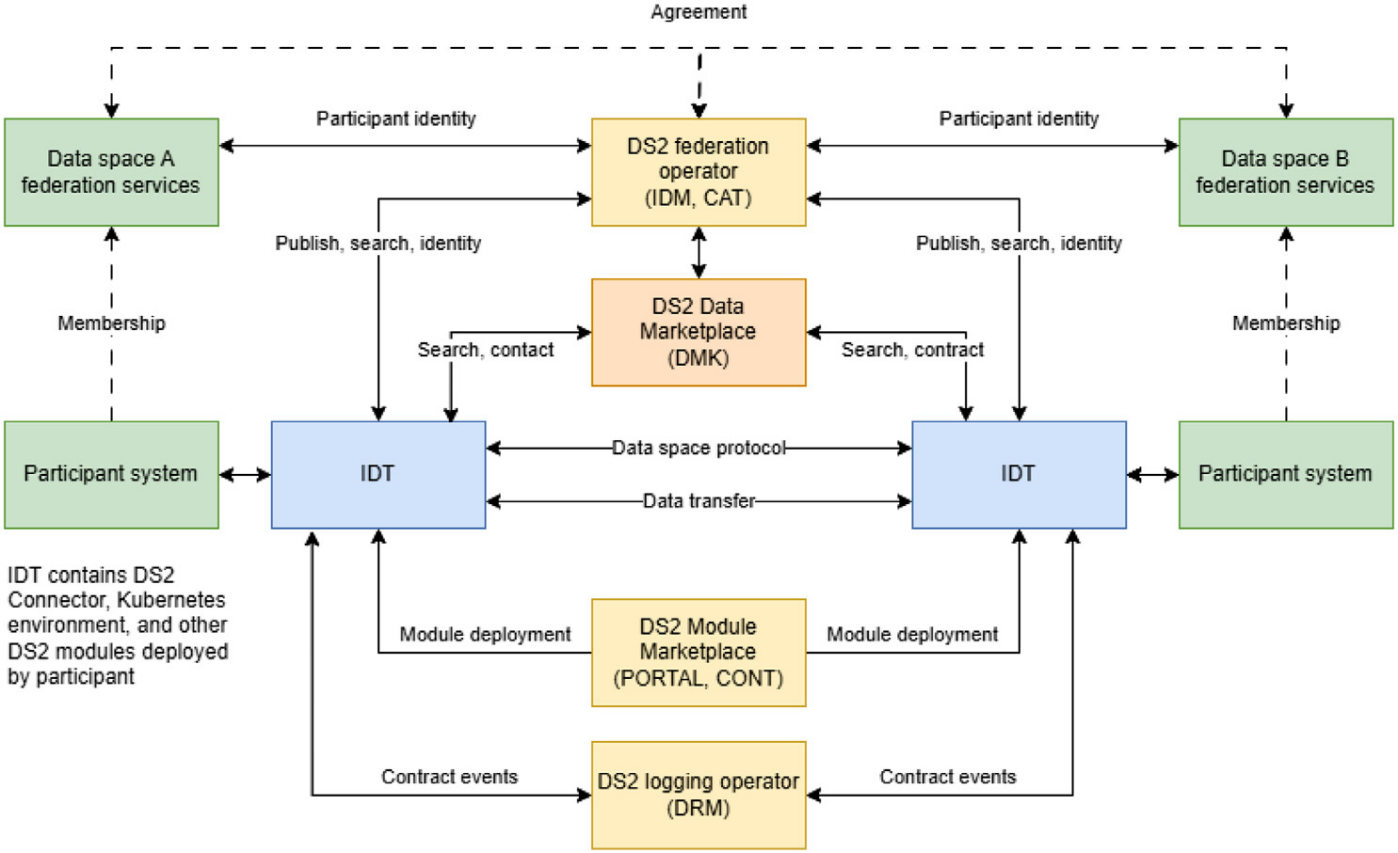
DS2 implementation of DSIA

An overview of DS2 deployment is given in Fig. 13. It is close to IDSA reference architecture, with the main difference being the role of the IDT Module, the inter-sector data space toolkit. In addition to hosting the DS2 connector, the IDT also provides the deployment and runtime environment for DS2 modules that can be used to extend the user's data management system.

The prerequisite for deployment is the DS2 infrastructure, i.e., federation services, logging services, and module marketplace services. These services can be hosted by one legal entity or distributed to several entities with the roles of DS2 operator, DS2 marketplace, and DS2 logging service. The IDM (identity management) and CAT (catalogue) modules are the technical backbone of the federation solution. The data offering catalogue and participant information services need CAT modules. The federation has additional services, such as participant management.

In terms of DS2 as a data space interoperability solution, the data spaces A and B need to exist, and there must be a collaboration agreement between them and the DS2 operator. In practice, this means the permission of the DS2 operator to expose the metadata from data spaces to each other and the acceptance of the DS2 operator as a service intermediary to those data spaces. This is needed to allow the DS2 IDM module to verify participants' identities from data spaces' identity verification services. The IDM module also manages the data space identities and data space agreement identities (data space pairs).

When data space participants join DS2, they are registered by the federation operator and granted access to the module marketplace (built using PORTAL and CONT modules), where they acquire and deploy the IDT module that contains the DS2 connector with valid credentials for trustworthy communication, CONT (a Kubernetes based runtime environment for containerised modules), and interface to the marketplace for further deployment of DS2 modules. The DS2 connector is extended with the DS2 PAE (policy enforcement) and CAT (participant's product offerings catalogue) modules. After this stage, the participants are ready to start inter-data space operations.

Participants can enhance their data processing, data management, and user experience capabilities by deploying additional DS2 modules from the module marketplace. A DSIA data pipeline can be facilitated by deploying the Orchestration module (ORC) orchestrating data pipeline modules such as Data Detection and Transformation (DTT), Data Curation (CUR), Data Inspector (DINS), and Data Share Controller (DSHARE) modules. Data transfer capabilities can be improved with edge-to-cloud (E2C), Multi-cloud (MCL), Security (SEC), and Data Retrieval (RET) modules. LLM-based chatbots in the DARC module and the culture and language module (CLM) enhance the ability to formulate queries to catalogues and interpret results, leading to more efficient searches and configurations. The Model Development Toolkit (MDT) module supports AI application development, while the Sovereignty Decision Support (SDS) module aids decision-making for collaboration across data spaces through risk modelling and analysis capabilities.

The list of current modules in DS2 is not fixed, and the module marketplace can include more modules in its portfolio. However, the current implementation does not support trustworthy module concepts, as this would require developments in the governance domain and the addition of issues such as module certification.

### 5.3 DS2 in use cases

The DS2 modules are mostly based on existing technologies and solutions validated in multiple use cases. The DS2 connector is based on the Eclipse data space connector. Kubernetes and Docker containers are widely used. Identity management is like existing services in data spaces and other identity management systems. Catalogues are widely used in data portals and data management systems.

The DS2 system is being tested in multi-data space use cases. Six data spaces will be made interoperable using the DS2 solution. All the data spaces are still in the early ramp-up phase, which means that the technical solutions have been defined and taken into use, but the governance parts are mostly implemented using manual and not fully defined processes. The data spaces will run in three locations; they will serve different sectors, and the number of participants during this evaluation will be between 3 and 20 in each data space. The locations will be in Greece, Slovenia, and Romania, and the sectors will be precision agriculture and food production, green deals, and smart city services. The main characteristics of data spaces are given in Table 1.

**Table 1:** Data spaces of use cases.

Name	Description	Location	Technology
SmartCity (Cluj Napoca)	City data space aiming to support applications for citizens, researchers, and policy makers on wide area of sectors in the city.	Romania	IDSA (DSIL connector)
ZeroNET	Data space for services focusing on understanding the emissions and pollution.	Romania	IDSA (DSIL connector)
Agrifood data space	Data space for managing and sharing IoT, satellite, agricultural, and environmental data.	Slovenia	ITC platform data space
SmartCity (Murska Sobota)	City data space for sharing sensor data, data from city assets, and refined environment analysis results for applications that increase awareness of e.g. air quality for citizens, SMEs, industry, and government	Slovenia	IDSA (DSIL connector)
DigiAgro	Data space for farmers to share and combine their crop data with other environment data for creation of recommendations for farmers, and status data for food industry.	Greece	IDSA (TrueConnector)
AgroScience	Data space for satellite and detailed weather forecasts for data analysts and agronomists for their services.	Greece	IDSA (DSIL connector)

**Fig. 14:**
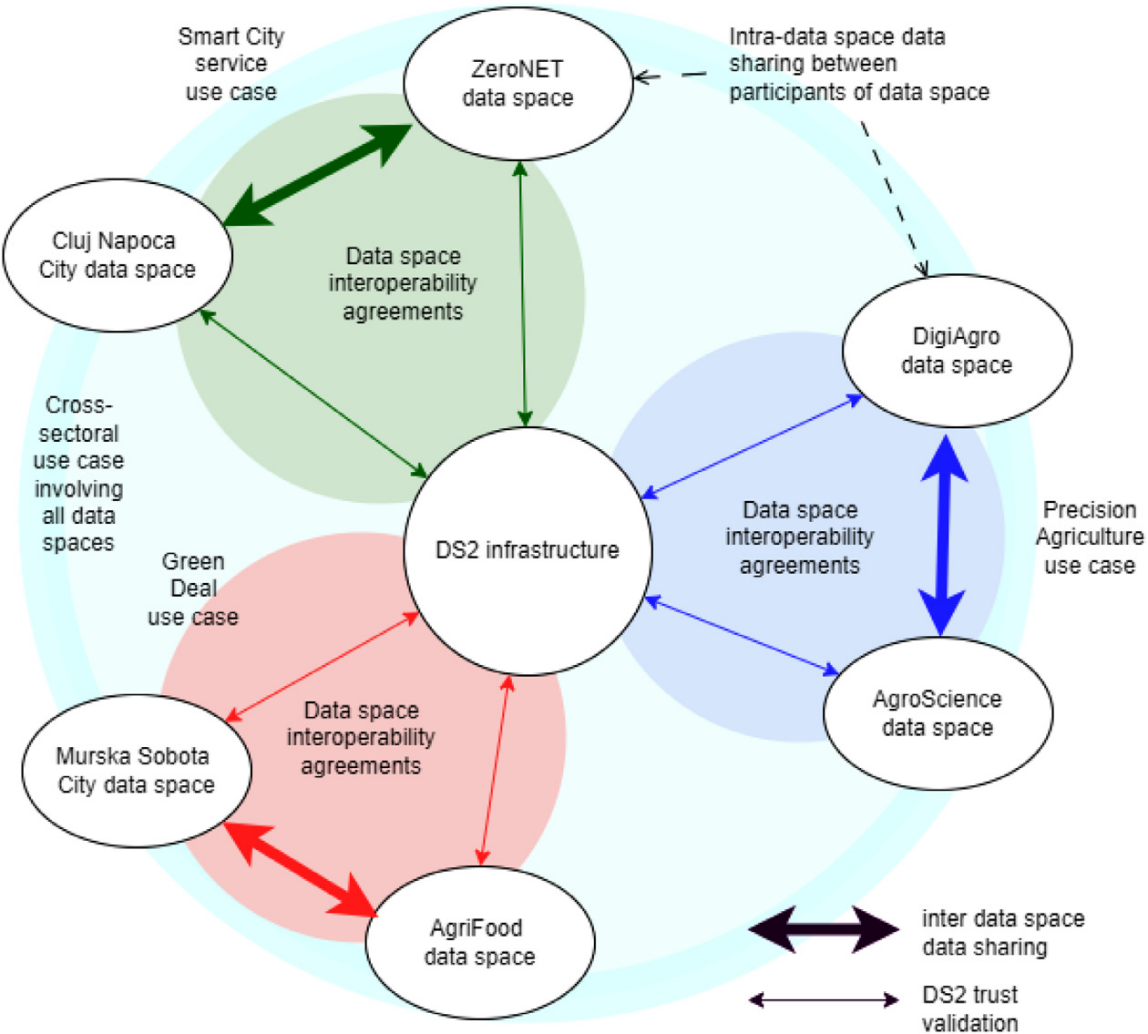
Overall architecture of use cases.

The overall architecture of DS2 use cases is given in Fig. 14. The interoperability of data spaces is implemented using the DS2 infrastructure services (IDM, CAT, DRM, IDT) and participant services (IDT) provided by the DS2 operator. The data spaces in all the pilots have agreements between themselves and the DS2 operator to use DS2 as an interoperability solution.

The participating organisations are members of these data spaces, and those who participate in inter-data space data sharing will also register to DS2 users as members of their home data spaces. They will install the DS2 IDT module and other DS2 modules each participant may need. During DS2 operations, the DS2 registration of data spaces and the validity of the collaboration agreement are verified from the DS2 IDM/PORTAL modules. DS2 Operator has a data space collaboration data base that contains information on agreements between data spaces. IDM verifies the participant's membership from the home data space's identity verification service. The DS2 CAT and DRM services are used in the same way as in a single data space use case, except that the CAT service restricts the visibility of offerings to those data spaces that have a collaboration agreement.

The data products shared in these cases come from various data producers, such as farmers, service providers, IoT and data platforms, etc. There are sensor data streams from farms and city environment, images ranging from micro cameras in farms to enriched satellite images, results from data analytic and AI services, city transportation information including real-time tracking of vehicles, real-time energy production and consumption data in citizen and commercial context, and many types of additional city and environment data sources. Each data provider/consumer has IDT with a DS2 connector and a set of data services providing access to the data. Technically, the data services in DS2 use cases are data sets accessed from web server or data base APIs. Data providers will create data offerings with data-sharing contracts published in the DS2 catalogue. Technically, data sharing across data space boundaries is performed as within a single data space. The difference between DS2 and creating another data space layer on top of existing ones is that in DS2, the collaboration is set up by data space governance authorities and the DS2 operator. The user, the data space participant, does not need to go through any complex onboarding processes with validations and acceptance of additional rules. It only needs to register to DS2 solution as a member of a data space, to provide the same participant information to DS2 as it has provided to its home data space, to acquire and deploy the IDT module with DS2 identities, and possibly other DS2 modules into its own server, and to start using it.

### 5.4 Experiences from use cases

As the DS2 approach utilises dedicated participant services, the primary differences in data sharing from the user's perspective are related to setting up collaboration between data spaces and establishing participant services. The actual data sharing operations are very similar to those that occur within a data space. The main findings are as follows.

**Setting up data space agreements:** The joining of a data space to the DS2 solution is facilitated through the DS2 web portal. Each data space fills in the basic information that is stored in the DS2 registry, and the DS2 Operator assigns an identity to the data space. It is a straightforward process that utilises a web service. In the use case data spaces, the governance implementations have services on participant management only. Therefore, the collaboration agreements between data spaces have been established manually and registered manually in the DS2 Operator registry.

**Joining DS2 and setting up IDT for data space participants:** Participants join DS2 through a web service. They fill in basic information, and the DS2 Operator checks that they are participants in their data spaces, which must have been joined to the DS2 Operator service beforehand. Participants are given identities and permissions to upload the IDT module from the DS2 operator module marketplace. The deployment of IDT, which includes the DS2 connector to the participant system, depends naturally on the participant system; however, in our experiments, it is an easy and straightforward process. IDT has its UI, but connecting participant system services to it and the DS2 connector is like connecting to any data space.

**Identity management:** In DS2 the DS2 Operator issues and verifies identities of participants, DS2 components, and collaboration agreements. In case of participants, it also verifies from the home data space that participant has an active membership. There are three options depending on the type of data space. In case of data spaces focused on sharing open data such as ZeroNeT and Agrifood data space, the identity verification may be open to everyone, and IDM must have an interface configured for it. In case of data space targeted to sharing of confidential B2B data, the identity verification may be limited to data space members. If data space supports identity verification for known intermediaries, the access to service can be granted as part of DS2-data space agreement. IDM naturally must support the communication with such interface. In other cases, the DS2 operator can join the data space as a member, deploy its data space connector and use that for identity verification. The VTT DIL data spaces that are based on IDSA RAM v3 architecture use this option. This option can be used in all data spaces, but it must be noted that the DS2 Operator’s connector is used only for identification purposes and not for actual data sharing.

**Publishing and searching data products:** The DS2 has its own data offer catalogue service, which is used in the same way as a standard data space catalogue. The only difference is that the data offer must be compliant with the DS2 data offer model, which is like DSSC recommendations; however, the catalogue itself is agnostic to the data model used. It is possible to modify and extend the data model if needed. Publishing and searching take place through the DS2 connector and its UI.

In the current DS2 implementation, it is not possible to publish a home data space data product in the DS2 catalogue or to create a data sharing contract across data spaces using the home data space data offer. The reason is separate participant services, i.e., the home data space connector and DS2 connector. Current connector implementations do not support the use in more than one data space per connector instance. Transforming the data product offer, for example, from the IDSA RAM v3 model to the newer IDSA RAM v4 model would be relatively easy.

**Data sharing in DS2:** Executing the data transaction is performed by the DS2 connector. In the use cases, the data services are basic HTTP services and simple database APIs. The DS2 connector's PAE extension and data plane are fully capable of implementing the necessary transactions. The EDC policy enforcement and DS2 PAE module are extensions of IDSA RAM v3 policies, so it would not be a restriction. Regarding the data plane, even the basic EDC and DS2 connector data plane is more capable than the IDSA v3 data space protocol. Both the data plane and policy enforcement can be extended with new functionalities. New data planes can be added, and new external policy enforcement functions can be connected to PAE, with respective policies added to data sharing contracts.

**Data pipeline and supporting functionalities:** The use cases will also demonstrate the data pipeline and supporting DS2 modules that aim to take the usability of data spaces to the next level. Examples include the use of a data pipeline as an extension to the data product definition, the risk analysis using SDS tools, chatbots using DARC and CLM modules for adding capabilities to improve data searches and data processing orchestration by going beyond the formal metadata and benefitting of advanced AI model development processes using DMT module. These modules are currently under development, but the DSIA architecture and its DS2 implementation provide means to integrate these capabilities into data spaces. As a summary of the experimentations in use cases, DS2 implements the basic operations required by the interoperability solution in our use cases. The used data spaces and use cases are only examples with very limited features and do not represent the full potential of the data space concept. At the same time, the governance functionalities are not specified in detail, and the implementations of governance may vary a lot. Providing extensive validation in such a situation is very difficult. The data space concept is very flexible and configurable as well. The types of data needed, data sharing contracts, policy enforcement functions, and data planes, among others, have numerous options that cannot be covered in any set of use cases. Being ready for changing needs to data sharing is a challenge for data spaces themselves, let alone for their interoperability solution. DS2 has built-in support for extending its capabilities, and the proposed interoperability solution focuses on the core functionality of data transactions. In that sense, it is prepared to adapt.

### 5.5 Comparison to another interoperability approaches

As described in Chapter 2.5, there are three main interoperability concepts: collaborating data spaces implemented using interlinking layer (Solution A), a federation of data spaces approach (Solution B), and an intermediary-based approach (Solution C). Comparing interoperability approaches is challenging because not all of them have published implementations. In addition, the concepts can be implemented in several ways, which affects their characteristics. Implementations may also combine elements from multiple approaches. We have created the following illustrative examples of alternatives for comparison purposes. In the alternatives, we have emphasised the trustworthy data transactions directly between participants, suitability to all kinds of data space technologies, and the capability to implement data space principles of sovereignty and trustworthiness.

Solution A, collaborating data spaces, is based on an interoperability-by-design approach, where all participating data spaces use interoperable services based on a common standard and set of solutions. The interoperability of data spaces extends interoperability within a data space. In practice, this limits collaboration to data spaces using the same technology or requires adding an interlinking layer with services. The governance model is a decentralised collaboration of data spaces.

Solution B is based on the federation of data space approach, where interoperability is achieved through an additional governance authority that defines how data spaces operate together. The governance authority hosts all federation services and provides participant services to all users. The governance model is centralised.

Solution C is represented by the DS2 intermediary approach, where a service provider hosts federation services that implement services like an interlinking layer between data spaces. DS2 does not have a governance structure in the sense of a data space. The operating rules come from collaboration agreements between participating data spaces.

Table 2 presents the analysis results. We have compared the solutions from technical, service, governance, legal, and business interoperability perspectives, implementation complexity, data space participant, and scalability perspectives.

**Table 2:** Comparison of DS2 to illustrative interoperability approaches.

	Solution A (Collaborating data spaces, interlinking layer)	Solution B (Federation of data spaces)	Solution C: Intermediary approach, DS2
Technical, semantic and syntactic interoperability	Common data models, protocols and APIs in federation and participant services.	The technical solutions are defined by common governance framework.	Based on additional common participant services.
Service interoperability issues	All federation services should be interoperable across data spaces.	All federation services are common, therefore, no need for interoperability across the federated data spaces.	The identity validation requests from interoperability federation service must be supported by data spaces.
Governance, legal and business interoperability	Defined by agreements between data spaces leading to decentralised governance.	The common governance framework defines everything. Overheads caused by governance.	Defined by agreements between federation interoperability service operator and data spaces. Third party overheads.
Implementation complexity of solution	All data spaces must be built on using compatible components.	Common additional participant services are needed. Common federation operator needed.	Components must be deployed in all participants systems and common federation interoperability service provider is needed
Data space participant perspective	Participant has automatic access to services after data space agreements	Participant of the data space can decide after data space has joined the data space collaboration.	Participant of the data space can decide after data space has joined the data space collaboration.
Scalability of data space network	Affects to all data spaces’ federation services as they must be able to serve all participants of all agreed data spaces.	Affects to mainly governance authority due to size of the federation.	Affects mainly to federation service provider who must manage agreements and federation service operations.

As shown in Table 2, all approaches can be used to create interoperability among data spaces. The difference lies in how complexity is distributed across the parts of the overall system and in the extent to which the integrity of data spaces and their partners is preserved. Solution A is the most integrated approach, distributing the complexity and governance load across participating data spaces and participants as needed. Additional services must be deployed to both. Solutions B and C introduce additional operators that reduce the load on the data spaces by providing federation services. The main difference between B and C is that B creates an additional data space layer on top of collaborating data spaces with its own rules, whereas C provides federation services. In all cases, the additional participant agent services are needed to ensure the point-to-point communication between participants.

## 6 Discussion

The data space concept has the potential to integrate data sources outside the organisation into organisation’s data management, to create a trustworthy data sharing environment, and to implement contract-based data transactions. Data space participants are sovereign companies that can decide by themselves what kind of collaboration they do with each other. However, if they base their collaboration and trustworthiness on being members of data spaces, then when dealing across data space it must be based on mutual understanding of both data spaces. The mutual understanding means that data spaces involved in collaboration must agree the governance principles between them a source of trust in data transactions. This does not mean the governance principles are identical; the keyword is acceptance. These collaboration agreements must be supported by the implementation of governance principles. There must be a registry related to collaboration agreements and data spaces must support the needed interactions of federation services related to interoperability functions. Current data space specifications should be extended to take these into account.

Technical interoperability sets the requirements for the implementation of collaborations. The first technical interoperability requirement is the ability to execute trustworthy data transactions between the participants. There must be interoperable participant services between participants of different data spaces and federation services that they can use.

In the DSIA and DS2, the dedicated solutions based on concepts recommended by the DSSC Blueprint [30] have been chosen, because interoperability between data spaces is an extension to data spaces, and similar solutions are a natural choice. The three strong arguments favouring the intermediary service approach and use of data space technologies are 1) similar trust mechanisms for components, 2) resilience against technical variations in components, and 3) minimal dependency on technology choices of participating data spaces. The counterargument is the need for parallel services to what is already available in data spaces. Participants need to host additional participant services and adapt their processes for operating with data spaces that include these services. There is also a need for service intermediaries that operate DSIA federation services at additional costs.

The alternatives to overcome the technical interoperability requirements would be as follows:

1. To force all data spaces to rely on interoperable technical solutions, which means, in practice, having identical data space protocols and federation services. The GAIA-X ecosystem is an example of this, and it can provide a simple, low-cost solution by separating data spaces with access rights and possibly differentiating operational rules. The challenges are the risk of being either too complex and expensive to deploy and maintain or too restrictive in their capabilities for space participants. Of course, it also prevents data spaces using different technologies from joining.
2. To have an intermediary that does all data and message transformations between data spaces. The intermediary would have data space-specific participant agent services for interaction with participants. This approach would use data spaces' own participant agent services, minimising the need to change anything on the participant side. The data space federation services should support the use of an intermediary as a proxy to an actual participant from another data space. The intermediary should implement the access management across data spaces and host and implement the collaboration rules between data spaces. The primary concern with the approach is that participant agents in data transactions cannot communicate directly with each other, so communication and data transfer must be routed through an intermediary, violating the point-to-point data transfer requirement of data spaces and adding risks to data sharing. Another concern is the complexity of transformations that increases with the number of different technologies and data spaces joining services.

Interoperability of trust is a more complex issue. Trust in data spaces is created through common operational principles, known identities, trustworthy components, and common legal frameworks implemented with data space agreements, membership, component certificates, and data-sharing contracts.

The DSIA approach leaves the acceptability of data space operational principles to data spaces and DSIA service operators to manage. Operational principles include onboarding processes, participant evaluations, and data space rules that cannot be transferred to technical solutions and require human intervention. When the acceptance exists, it must be recorded in data space interoperability agreements, and its validity ideally should be verified when participants act. In the DS2 this is done by the DS2 Operator whose technical governance solution must support it.

With known identities and membership, the DSIA relies on the membership of participating data space as a source of trust. This is the only governance issue requiring collaboration between the DSIA and data space services. The alternative would be to create their own DSIA identities for participants, which should include evaluating participants and monitoring their status in participating data spaces, which would be at least equally complex as a chosen approach.

The trustworthiness of components could be based on data spaces' own certificates, but as DSIA is based on dedicated services, it needs its own component identities to be verified internally. Ideally, the software component identities should be separated from the data space governance, as this would allow for the use of components in multiple data spaces simultaneously. The separation would require implementing independent certification authorities for components and verification services for those certificates. In cases where two data spaces use similar components, the use of separate participant services could be at least partially avoided.

Data-sharing contracts are part of trustworthy data transactions. However, they can include rules that come from data spaces' operational principles, and the extent to which they are applied to inter-data space data sharing should be defined by participating data spaces. This may lead to very complex combinations of rules that must be applied to different data transactions based on what data space interoperability agreement is the basis of collaboration. Legal and business issues should be treated similarly. In the ideal case, the interoperability solution provider should create a governance structure that includes operational principles to all data space interoperability agreements separately as they may differ from each other, and there should be a service that adds the needed contract conditions to initial data sharing agreements depending in which data spaces are participating. Another extreme is that inter-data space data sharing agreements can be made without any data space-related rules and constraints, and anything between these two is also possible. DSIA has adopted a solution: the interoperability solution provider must maintain its operational principles, but what and how it is implemented is left open.

## 7 Conclusions

Data spaces have evolved into trustworthy environments for contract-based trusted data transactions, and the first data space solutions have started their operations. The use cases for data spaces are diverse and have different requirements that are not feasible to solve with a single unified approach. The specifications and standards have not matured yet, leaving opportunity for dedicated solutions. The data space interoperability architecture (DSIA) and its DS2 implementation provide a solution for participants of different data spaces to exchange data based on their trustworthiness as data space members. DSIA is based on interoperability services for participants and data space governance authorities. The use of the services is based on mutual understanding and acceptance of governance principles of the involved data spaces.

Both DSIA and its DS2 implementation rely on loosely coupled federation services built using technologies recommended by the DSSC. To support a large variety of data spaces, the DSIA has a minimal dependency on the technology choices of participating data spaces. Whilst this approach requires additional participant services and service intermediaries, it avoids the complexity and expense of forcing all data spaces to rely on same technical solutions. The deployment of DS2 to participant systems is based on an inter-sector toolkit (IDT) module that includes Kubernetes-based containerised run-time environment, a dedicated Eclipse Connector-based DS2 participant services, and support to install additional containerised services and applications from the DS2 portal and marketplace. To help with data transformations, analyses, and processing, DS2 includes a data pipeline orchestration mechanism, where data pipelines can be created and executed from modules deployed from the DS2 marketplace.

The DSIA and DS2 have been compared against interoperability-by-designs and federated data spaces concepts. They avoid technology limitations and provide more flexibility than a common governance framework. Costs are associated with additional interoperability federation services or interfaces with legacy data spaces. The DS2 implementation takes steps towards making data spaces an extension to the company’s data management by providing holistic user interfaces and applications that support users in their attempts to exploit external data sources efficiently.

## Ethics Statement

The authors have read and follow the ethical requirements for publication in Data in Brief and confirm that the current work does not involve human subjects, animal experiments, or any data collected from social media platforms.

## CRediT authorship contribution statement

**Juha-Pekka Soininen:** Writing – original draft, Conceptualization, Methodology. **Carlos Fernández Sánchez:** Software, Conceptualization, Writing – review & editing. **Stefano Modafferi:** Conceptualization, Writing – review & editing. **Stuart Campbell:** Conceptualization, Writing – original draft, Funding acquisition. **Noel Tomas:** Software, Conceptualization, Writing – review & editing. **Eliot Salant:** Conceptualization, Writing – review & editing. **Christina Manara:** Software, Writing – review & editing. **Jarmo Kalaoja:** Software, Writing – review & editing. **Soumya Kanti Datta:** Software, Writing – original draft.

## Footnotes

1 DS2 approach is a data space interoperability architecture and solution developed in EU Data Space, Data Sharing 2.0 project. The work is published at www.github.com/ds2-eu/architecture [52].

## References

[1] “11 Insightful Statistics on Data Market Size and Forecast | Edge Delta.” (Accessed 18 December 2024). [Online]. Available: https://edgedelta.com/company/blog/data-market-size-and-forecast

[2] European Parliament and Council of the European Union (2016). https://eur-lex.europa.eu/eli/reg/2016/679/oj.

[3] European Parliament and Council of the European Union (2023). https://eur-lex.europa.eu/eli/reg/2023/2854/oj.

[4] European Parliament and Council of the European Union (2022). https://eur-lex.europa.eu/legal-content/EN/TXT/?uri=CELEX:32022R0868.

[5] Curry E. (2020).

[6] Otto B., ten Hompel M., Wrobel S. (2022). Designing Data Spaces: The Ecosystem Approach to Competitive Advantage.

[7] Curry E., Scerri S., Tuikka T. (2022).

[8] “DS2 | Dataspace | Data Share.” (Accessed 13 May 2025). [Online]. Available: https://www.dataspace2.eu/

[9] International Data Spaces Association (IDSA) (2021). https://dataspaces.radar.eu.

[10] Punter M. (2023). https://resolver.tno.nl/uuid:7c857422-981f-440a-8780-4a08b0e2b39d.

[11] European Commission (2024). https://ec.europa.eu/info/strategy/priorities-2019-2024/europe-fit-digital-age/european-data-strategy_en.

[12] Data Spaces Support Centre (2025). https://dssc.eu/space/BVE2/1071252241/Cross-data+space+interoperability+considerations+in+data+space+design+and+operation.

[13] File Transfer Protocol (1971). https://www.rfc-editor.org/info/rfc114.

[14] Luidold C., Jungbauer C. (2024). Cybersecurity policy framework requirements for the establishment of highly interoperable and interconnected health data spaces. Front. Med..

[15] von Scherenberg F., Hellmeier M., Otto B. (Feb. 2024). Data sovereignty in information systems. Electron. Markets.

[16] Depa V.R. (2025). The evolution of API management: transforming modern integration landscapes. Int. J. Comput. Eng. Technol..

[17] Yang Q., Ge M., Helfert M. (2019). 21st International Conference on Enterprise Information Systems (ICEIS).

[18] Deepa N. (2022). A survey on blockchain for big data: approaches, opportunities, and future directions. Future Generation Computer Systems.

[19] Franklin M., Halevy A., Maier D. (2005). From databases to dataspace: a new abstraction for information management. SIGMOD Rec..

[20] Halevy A., Franklin M., Maier D. (2006). *Proceedings of the Twenty-Fifth ACM SIGMOD-SIGACT-SIGART Symposium on Principles of Database Systems*, in PODS ’06.

[21] Bacco M., Kocian A., Chessa S., Crivello A., Barsocchi P. (2024). What are data spaces? Systematic survey and future outlook. Data Br..

[22] Sitra (2022). https://www.sitra.fi/en/publications/rulebook-for-a-fair-data-economy/.

[23] Nagel L., Lycklama D. (2021). Design principles for data spaces - position paper. Zenodo.

[24] International Data Spaces Association (2022). https://github.com/International-Data-Spaces-Association/IDS-RAM_4_0.

[25] Gaia-X (2022). https://docs.gaia-x.eu/technical-committee/architecture-document/22.10/.

[26] “Data Spaces Support Centre.” (Accessed 18 December 2024). [Online]. Available: https://dssc.eu/page/about

[27] Data Spaces Support Centre (2024). https://dataspace.eu/blueprint-v2.0.

[28] European Commission (2024). https://commission.europa.eu/document/swd2024_21_final.

[29] “Simpl: Cloud-to-edge federations empowering EU data spaces | Shaping Europe’s digital future.” (Accessed 19 December 2024). [Online]. Available: https://digital-strategy.ec.europa.eu/en/policies/simpl

[30] Data Spaces Support Centre (2024). https://dataspace.eu/blueprint-v2.0.

[31] Hutterer A., Krumay B., Muehlburger M. (2023). AMCIS 2023 Proceedings.

[32] J.-P. Soininen et al., “Data spaces’ synergies.” Data Space Support Centre c/o Fraunhofer-Gesellschaft zur Förderung der angewandten Forschung e. V., Munich, Germany, 2024. [Online]. Available: https://dssc.eu/space/DSSE/758350768/Data+Spaces'+Synergies

[33] “Smart Connected Supply Network.” (Accessed 4 March 2025). [Online]. Available: https://smart-connected.nl/en

[34] “Catena-X Your Automotive Network | Catena-X.” (Accessed 4 March 2025). [Online]. Available: https://catena-x.net/en/1

[35] “sovity: Sovereign data exchange with your partners in Data Spaces.” (Accessed 4 March 2025). [Online]. Available: https://sovity.de/en/sovity-en/#pll_switcher

[36] “DataSpace Europe.” (Accessed 4 March 2025). [Online]. Available: https://www.dataspace.fi/en/homepage

[37] (2025). https://dataspace.dih-agrifood.com/.

[38] Soininen J.-P., Laatikainen G. (2025). What is a data space—logical architecture model. Data Br..

[39] Nambiar A., Mundra D. (2022). An overview of data warehouse and data lake in modern enterprise data management. Big Data Cogn. Comput..

[40] Serhani M.A., El-Kassabi H.T., Shuaib K., Navaz A.N., Benatallah B., Beheshti A. (2020). Self-adapting cloud services orchestration for fulfilling intensive sensory data-driven IoT workflows. Fut. Gener. Comput. Syst..

[41] Interoperable Europe Programme (2024). https://interoperable-europe.ec.europa.eu/collection/semic-support-centre/solution/dcat-application-profile-data-portals-europe/release/300.

[42] W3C (2023). https://www.w3.org/TR/odrl-model/.

[43] W3C (2023). https://www.w3.org/TR/vc-data-model/.

[44] CEN-CENELEC (2024). https://www.cencenelec.eu/media/CEN-CENELEC/CWAs/RI/2024/cwa18125_2024.pdf.

[45] “Dataspace Protocol 2024-1 | IDS Knowledge Base.” (Accessed 4 March 2025). [Online]. Available: https://docs.internationaldataspaces.org/ids-knowledgebase/dataspace-protocol

[46] E. Commission, D.-G. for Mobility, Transport, VTT, Ricardo, and Wavestone (2025).

[47] (2025). Gaia-X European Association for Data and Cloud AISBL, Brussels, Belgium.

[48] Crnkovic G.D., Magnani L., Carnielli W., Pizzi C. (2010). Model-Based Reasoning in Science and Technology: Abduction, Logic, and Computational Discovery.

[49] Mayer R.C., Davis J.H., Schoorman F.D. (1995). An integrative model of organizational trust. Acad. Manag. Rev..

[50] Rousseau D.M., Sitkin S.B., Burt R.S., Camerer C. (1998). Not so different after all: a cross-discipline view of trust. Acad. Manag. Rev..

[51] W3C (2023). https://www.w3.org/TR/odrl-model/.

[52] DS2-EU (2025). https://github.com/ds2-eu/architecture.

[53] “Eclipse Dataspace Components.” (Accessed 4 March 2025). [Online]. Available: https://github.com/eclipse-edc/Connector

